# Eosinophils Decrease Pulmonary Metastatic Mammary Tumor Growth

**DOI:** 10.3389/fonc.2022.841921

**Published:** 2022-06-08

**Authors:** Rachel A. Cederberg, Sarah Elizabeth Franks, Brennan J. Wadsworth, Alvina So, Lisa R. Decotret, Michael G. Hall, Rocky Shi, Michael R. Hughes, Kelly M. McNagny, Kevin L. Bennewith

**Affiliations:** ^1^ Integrative Oncology, BC Cancer, Vancouver, BC, Canada; ^2^ Pathology and Laboratory Medicine, University of British Columbia, Vancouver, BC, Canada; ^3^ Interdisciplinary Oncology Program, University of British Columbia, Vancouver, BC, Canada; ^4^ Biomedical Research Centre, University of British Columbia, Vancouver, BC, Canada; ^5^ Medical Genetics, University of British Columbia, Vancouver, BC, Canada

**Keywords:** eosinophil, lung metastasis, breast cancer, EO771, degranulation, interleukin-5

## Abstract

Metastatic breast cancer is challenging to effectively treat, highlighting the need for an improved understanding of host factors that influence metastatic tumor cell colonization and growth in distant tissues. The lungs are a common site of breast cancer metastasis and are host to a population of tissue-resident eosinophils. Eosinophils are granulocytic innate immune cells known for their prominent roles in allergy and Th2 immunity. Though their presence in solid tumors and metastases have been reported for decades, the influence of eosinophils on metastatic tumor growth in the lungs is unclear. We used transgenic mouse models characterized by elevated pulmonary eosinophils (IL5Tg mice) and eosinophil-deficiency (ΔdblGATA mice), as well as antibody-mediated depletion of eosinophils, to study the role of eosinophils in EO771 mammary tumor growth in the lungs. We found that IL5Tg mice exhibit reduced pulmonary metastatic colonization and decreased metastatic tumor burden compared to wild-type (WT) mice or eosinophil-deficient mice. Eosinophils co-cultured with tumor cells *ex vivo* produced peroxidase activity and induced tumor cell death, indicating that eosinophils are capable of releasing eosinophil peroxidase (EPX) and killing EO771 tumor cells. We found that lung eosinophils expressed phenotypic markers of activation during EO771 tumor growth in the lungs, and that metastatic growth was accelerated in eosinophil-deficient mice and in WT mice after immunological depletion of eosinophils. Our results highlight an important role for eosinophils in restricting mammary tumor cell growth in the lungs and support further work to determine whether strategies to trigger local eosinophil degranulation may decrease pulmonary metastatic growth.

## Introduction

Breast cancer is the second most common cancer among Canadian women, and patients diagnosed with stage IV breast cancer have a dismal survival rate, largely due to metastatic disease (1). The metastatic tumor microenvironment is a complex ecosystem, hosting a variety of pro- and anti-tumorigenic immune cells. These host immune cells play a dynamic role in both supporting tumor progression and coordinating innate and adaptive immune responses that can result in anti-tumor immunity. Certain cytotoxic immune cell subtypes, such as CD8^+^ T cells and NK cells, have been extensively characterized within primary tumors and metastases. Similarly, we and others have established the influence of immune suppressive myeloid-derived suppressor cells (2), macrophages (3), and regulatory T cells (4) on metastatic tumor growth in distant tissues. However, our understanding of how other immune cells that are present in metastatic target organs affect metastatic tumor growth is lacking. Improving our understanding of less well-characterized immune cells, such as eosinophils, may lead to the development of novel therapeutics to enhance anti-tumor immunity at metastatic sites.

Eosinophils are a population of granulocytic innate immune cells that are largely associated with asthma, allergy, and type 2 immunity (Th2). Eosinophils develop from myeloid precursors located in the bone marrow in response to the cytokines interleukin-3 (IL-3), granulocyte-macrophage colony-stimulating factor (GM-CSF), and IL-5, which each signal through the beta common chain of the IL-3 receptor. Following bone marrow egress, IL-5 remains a key cytokine for eosinophil migration/chemokinetic activity, survival, and activation (5). The majority of eosinophil research has focused on the ability of eosinophils to target and kill parasites, viruses, and bacteria *via* the release of cytotoxic granule proteins, including major basic protein (MBP), eosinophil peroxidase (EPX), eosinophil cationic protein (ECP), and eosinophil-derived neurotoxin (EDN) (6). However, emerging evidence demonstrates that eosinophils also participate in homeostatic processes, such as tissue remodeling and vascularization (7, 8). Although the presence of eosinophils in primary tumors and metastases was first reported over one hundred years ago (9–11), the potential role of eosinophils in tumorigenesis remains controversial.

Similar to other innate immune cells, such as macrophages and neutrophils, the role of eosinophils within the tumor microenvironment likely depends on polarizing factors secreted by both cancer cells and host cells, and direct interactions with the microenvironment. In this context, it is intriguing that eosinophil infiltration into primary tumors can be a negative or positive prognostic factor. For example, tumor eosinophil infiltration correlates with improved overall survival in colorectal cancer (12, 13), whereas eosinophil tumor infiltrates are associated with decreased overall survival and increased invasion in cervical cancer patients (14, 15). In preclinical models, eosinophils have been shown to increase bone marrow metastasis by CCR1^+^ melanoma cells directly, *via* secretion of chemotactic CCL6 (16), and indirectly *via* recruitment of immunosuppressive T regulatory cells (17) and dampening of NK cell function (18). However, a number of studies have emerged in the last decade demonstrating that eosinophils can alter the tumor microenvironment to improve immune checkpoint blockade efficacy (7, 8), modulate the phenotype and effector function of intra-tumoral immune cells (19), and kill tumor cells *ex vivo (* 20–23). Although recent studies have provided new insights into the complex role of eosinophils within the primary tumor, the role of eosinophils in the metastatic process is poorly understood.

The lungs are one of the most common sites of breast cancer metastasis, and we hypothesized that eosinophils in the lungs can decrease tumor cell colonization and metastatic growth. We used transgenic mouse strains with phenotypes displaying either elevated IL-5 and systemic eosinophilia (IL5Tg) or eosinophil-deficiency (ΔdblGATA) to determine the role of eosinophils in the colonization and growth of metastatic mammary tumor cells in the lungs of immunocompetent mice. Additionally, we assessed the tumor cell killing potential of eosinophils *ex vivo*, and determined whether degranulating eosinophils can be detected adjacent to tumor cells *in vivo*. We found that eosinophils can limit pulmonary metastatic colonization in eosinophilic IL5Tg mice, and that eosinophils in wild-type mice become activated over time and restrict the growth of tumor metastases in the lungs. Our data suggest that developing therapeutics to harness the anti-tumorigenic abilities of these innate immune cells could serve as a potential treatment option for metastatic disease.

## Materials and Methods

### Mice

Female IL5Tg, delta-double Gata (ΔdblGATA), ΔdblGATA/IL5Tg (Δdbl-IL5Tg) and wild-type (WT) C57Bl/6J mice (8-16 weeks old; Biomedical Research Centre, University of British Columbia, Vancouver) were maintained in micro-isolator cages on ventilated racks under specific-pathogen free conditions, housed in a modified barrier facility in the Animal Resource Centre at the BC Cancer Research Institute. Littermates were utilized for all experiments and mice were co-housed to control for potential microbiota-dependent effects on eosinophil phenotype and/or tumor growth. Animal experiments were performed in compliance with requirements of the Canadian Council on Animal Care and the University of British Columbia Animal Care Committee.

### Cell Lines

EO771 cells (CH3 Biosystems) were maintained in RPMI 1640 with 10% FBS. pLenti CMV GFP Blast (659-1) was a gift from Eric Campeau & Paul Kaufman (Addgene plasmid # 17445; http://n2t.net/addgene:17445; RRID : Addgene_17445) (24) and was used to generate EO771-GFP cells *via* lentiviral transfection. Lewis lung carcinoma (LLC) cells were generously provided by Dr. Gerry Krystal (BC Cancer) and maintained in DMEM with 10% FBS and 1x GlutaMAX. Mouse embryonic fibroblasts (MEFs) were provided by Dr. Nika Shakiba (UBC) and maintained in DMEM with 10% FBS + 1x GlutaMAX. MEFs were generated from WT C57Bl/6J mice housed in the Biomedical Research Centre at the University of British Columbia. To directly seed the lungs, 5x10^5^ EO771 or EO771-GFP cells were injected intravenously (i.v.) into the lateral tail vein.

### Tissue Processing and Serum IL-5 Quantification

Lungs were finely minced with scalpels and agitated for 40 minutes at 37°C with either 1mg/mL Collagenase Type II (Gibco Life Technologies) or 0.5U/mL Dispase Neutral Protease (Worthington), 100ug/mL Liberase (Roche), and 50μg/mL DNase (Sigma-Aldrich) in serum-free RPMI 1640. After tissue digestion, lungs were filtered through a 100µm cell strainer to create a single-cell suspension. For isolation of leukocytes from bone marrow and spleen for flow cytometry, femurs were flushed with PBS and spleens were passed through an 100µm cell strainer, followed by a 40µm cell strainer to generate a single cell suspension. Peripheral blood was collected *via* cardiac puncture following euthanasia into an EDTA-coated tube and erythrocytes were lysed with ACK lysing buffer (ThermoFisher Scientific). For serum isolation, blood was stored at 4°C in the absence of an anticoagulant and serum was collected following microcentrifuge. Serum IL-5 was quantified using a Mouse IL-5 ELISA Kit (R&D Systems).

### *Ex Vivo* T Cell Stimulation

Lungs were digested with 1mg/mL Collagenase Type II as previously described to generate a single cell suspension. Lung cells were plated in a v-bottom plate at 1x10^6^ cells/well in RPMI + 10% FBS + 1x PenStrep (Gibco Life Technologies) in the presence of 3μg/mL Brefeldin A (eBioscience) +/- Cell Stimulation Cocktail (eBioscience) containing PMA (81nM final concentration) and ionomycin (1.34μM final concentration). Cells were incubated at 37°C for 3 hours prior to flow cytometric analysis. 

### Flow Cytometry

Single-cell suspensions from peripheral blood, bone marrow, spleen, and lungs were washed with PBS and stained for 30 minutes on ice with eFluor^®^ 780 fixable-viability dye (eBioscience). Propidium iodide (PI) was used as a viability dye in flow panels that did not require intracellular staining and was applied following primary staining. Cells were washed and resuspended in Hanks’ balanced salt solution with 10mM HEPES (StemCell Technologies) + 2% FBS + 0.05% NaN_3_. Anti-murine CD16/32 (clone 2.4G2, eBioscience) was used to block cells prior to antibody staining. Cells were stained with the following antibodies on ice for 15 minutes: CD45-APC, CD8α-FITC, CD11b-PECy7, CD3ϵ-PE, CD43-FITC (eBioscience), Siglec-F-PE or Siglec-F-TexasRed (BD Biosciences), Siglec-F-BV421, Gr1-FITC, MHCII-BV500, CD11c-BV605, CD25-APC, CD4-BV605, CD11b-APC, CD19-PECy7, CD63-APC, CD69-BV711, and CD35/CD21-BV500 (Biolegend). For immune cell profiling, cells were fixed and permeabilized for 30 minutes using the FoxP3/Transcription Factor Buffer Staining Kit (eBioscience), followed by intracellular staining for FoxP3 (FoxP3-PECy7 or FoxP3-V421, Biolegend) for 1 hour. For ex vivo T cell stimulation, cells were fixed and permeabilized for 20 minutes using the Intracellular Fixation and Permeabilization Buffer Set (eBioscience), followed by intracellular staining with IFNγ-APC and TNFα-PE-Cy7 (Biolegend) for 1 hour. All samples were acquired on a BD LSRFortessa (FACSDiva software, BD) and analyzed with FlowJo (TreeStar). Immune cell populations were identified as follows: eosinophils (CD45^+^Gr-1^lo^CD11b^+^CD11c^lo^Siglec-F^+^), alveolar macrophages (CD45^+^Gr-1^lo^CD11b^-^CD11c^+^Siglec-F^+^), neutrophils (CD45^+^Gr-1^hi^CD11b^+^Siglec-F^-^), B cells (CD45^+^CD19^+^), CD8 T cells (CD45^+^CD19^-^CD4^-^CD8^+^), CD4 T cells (CD45^+^CD19^-^CD4^+^CD8^-^), T regulatory cells (CD45^+^CD19^-^CD4^+^CD8^-^FoxP3^+^).

### Immunohistochemistry

For tumor burden quantification, lungs were inflated and fixed with 10% formalin for 24 hours. Lungs were then washed and stored in 70% EtOH prior to tissue processing. Immunohistochemistry (IHC) and hematoxylin and eosin (H&E) staining were completed at the Deeley Research Centre (BC Cancer, Victoria, BC). Tissues were processed, paraffin-embedded, sectioned, and stained with H&E, followed by step sections at 150µm intervals for a total of 5 sections. Lung tumor nodules were quantified using ImageJ. mMBP-1 (rat mAb 14.7.4) was generously provided by Drs. James Lee and Elizabeth Jacobsen and was used to identify eosinophils *via* IHC. For IHC, 4µm sections were stained with a Biocare intelliPATH autostainer at room temperature. For the remaining procedure, all reagents were purchased from Biocare Intermedico (Markham, Ontario) unless stated otherwise. Samples were blocked with Peroxidased-1 and Rodent Block M prior to staining with the GFP (Cell Signaling Technology, cat#2555) and MBP cocktail diluted with Da Vinci Green. Following primary staining, sections were incubated with Rabbit Mach2 AP polymer and Rat 1-step HFP polymer (Abcam), followed by Warp Red chromogen, IP DAB chromogen, and Hematoxylin. Slides were scanned with a MoticEasyScan slide scanner and images were acquired through Aperio ImageScope.

### Tumor Cell Cytotoxicity Assay

Spleen and lungs were isolated from naïve IL5Tg mice as a source of eosinophils and single cell suspensions were generated as previously described. Red blood cells were lysed for 5 minutes at room temperature with ACK lysing buffer (ThermoFisher Scientific). Eosinophils were isolated from spleens using an anti-PE magnetic bead separation kit (Stem Cell Technologies) *via* depletion with CD19-PE and CD3ϵ-PE antibodies, and purity was confirmed by flow cytometry. Eosinophils were also isolated from both spleens and lungs using the same kit *via* Siglec-F-PE positive selection. 4x10^4^ EO771-GFP or LLC cells were seeded in a 96-well v-bottom plate and eosinophils were added at varying seeding ratios. In co-cultures containing MEFs, 1x10^4^ MEFs were seeded as previously described and eosinophils were added at a 1:10 T:E ratio. Co-cultures were incubated for 6, 12, or 24 hours, stained with 0.2ug/mL propidium iodide (PI) to identify dead tumor cells, and samples were collected *via* flow cytometry. Assays performed with LLC cells and MEFs were also stained with CD45-APC to gate out eosinophils prior to detecting PI^+^ tumor cells and MEFs. For quantification of tumor necrosis factor-α (TNFα), interferon-γ (IFNγ), and Granzyme B, culture supernatants were collected from splenic eosinophils co-cultured with EO771-GFP tumor cells at a 1:40 tumor cell-to-eosinophil target-to-effector cell (T:E) ratio. As per the manufacturer’s instructions, TNFα, IFNγ, and Granzyme B were all quantified *via* ELISA (R&D Systems).

### EPX Activity Assay

The *o*-phenylenediamine (OPD) method (25, 26) was adapted to measure peroxidase activity as a surrogate for EPX release from eosinophils in response to incubation with tumor cells. Tumor cell eosinophil co-cultures were established as previously described at either a 1:10, 1:20, or 1:40 T:E ratio in phenol red-free RPMI 1640 (Invitrogen) and incubated for 6 hours. Supernatants (80μL) were collected *via* centrifugation and incubated with an equal volume of *o*-phenylenediamine reagent (800μL 5mM *o*-phenylenediamine + 4mL 1M Tris (pH8) + 5.2mL H_2_O + 1.25μL 30% H_2_O_2_) for 30 minutes. The reaction was terminated with 80μL of 4M H_2_SO_4_ and absorbance was read at 492nm. To calculate % EPX activity, control wells containing only eosinophils were lysed prior to the collection of supernatants to determine total intracellular EPX content. Data are reported as a percentage of total EPX calculated as follows: [(absorbance of sample – absorbance of control eosinophils) x 100/absorbance of lysed wells]. Data are reported as the mean of 3-6 independent experiments.

### Pharmacological Eosinophil Depletion

To deplete eosinophils, mice were treated *via* intraperitoneal injection with 0.6mg/kg anti-Siglec-F (238047, R&D Systems) or IgG_2A_ isotype control (54447, R&D Systems). WT mice were treated one day after i.v. EO771 injection, and every 5 days thereafter. Mice were euthanized at various timepoints after i.v. injection *via* CO_2_ asphyxiation.

### Statistics

All statistical analyses were conducted in GraphPad Prism. A Shapiro-Wilk test was used to determine normality of data. For normally distributed data, two-tailed Student’s t-tests were used to compare means of two groups and a one-way ANOVA was used for immune profiling data when more than two genotypes were present in the data set. For tumor burden analysis of more than two genotypes, two-tailed Student’s t-tests were used, followed by the Benjamini-Hochberg procedure to control for false discoveries (Q=1). When appropriate, a paired two-tailed Student’s t-test was used. A non-parametric Kruskal-Wallis test was used to compare means between multiple groups lacking a normal distribution. To identify outliers, both a ROUT test (Q=0.2) and a Grubbs test were used. GraphPad Prism was also used to plot the data. Unless otherwise stated, all data are reported as mean ± SEM.

## Results

### Pulmonary EO771 Mammary Carcinoma Growth Is Reduced in IL5Tg Mice

**Figure 1:**
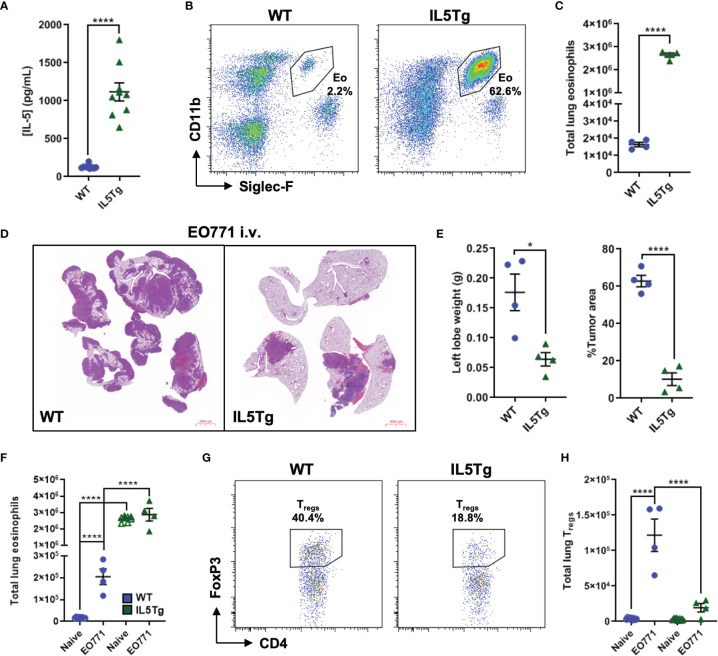
Pulmonary EO771 mammary carcinoma growth is reduced in IL5Tg mice. **(A)** Quantification of naïve WT and IL5Tg serum IL-5. **(B)** Representative flow plots of eosinophils (Eo) as a proportion of CD45^+^ cells in the lungs of naïve WT and IL5Tg mice. **(C)** Total number of eosinophils in the left lung lobe of naïve WT and IL5Tg mice. **(D)** Representative images of four right lung lobes harvested from WT and IL5Tg mice 21 days after i.v. injection of EO771 cells. Scale bar = 2000μm. **(E)** WT and IL5Tg left lung lobe weights and EO771 tumor area quantification as a proportion of total area of right lung lobes. **(F)** Quantification of eosinophils in the left lung lobe of naïve WT and IL5Tg mice and mice injected i.v. with EO771 cells 21 days post-injection. **(G)** Representative flow plots of T_regs_ as a proportion of CD4^+^ T cells in the left lung lobe of WT and IL5Tg mice 21 days after i.v. injection of EO771 cells. **(H)** Number of T_regs_ in the left lung lobe of naïve and EO771 i.v. injected WT and IL5Tg mice. Data points are individual mice with mean ± SEM of 4-8 mice per group. *p < 0.05; ****p < 0.0001.

Due to the critical role IL-5 plays in eosinophil differentiation, proliferation, and activation, IL5Tg mice that overexpress the cytokine IL-5 *via* a CD3δ-driven promoter (27) have been used to study eosinophils in a variety of different disease models (5, 11, 16, 27). Consistent with previous reports (27, 28), we found that naïve IL5Tg mice bred and housed at our facility have a 10-fold increase in the level of circulating IL-5 (**Figure 1A**) and a large increase in the number of eosinophils in the lungs (**Figures 1B, C**), blood, bone marrow (BM), and spleen (**Supplemental Figure 1A**).

To determine if systemic IL-5 over-expression alters pulmonary metastatic tumor growth, we utilized an experimental model of pulmonary metastasis by injecting EO771 mammary carcinoma cells intravenously (i.v.) to directly seed the lung tissue and harvested lungs for analysis 21 days following i.v. injection. We found that IL5Tg mice had significantly less pulmonary EO771 tumor burden than WT mice as indicated by decreased weight of the left lung lobe and reduced tumor area as a proportion of lung area (**Figures 1D, E**). We were also interested in potential differences in the immune cell infiltrate in the lungs of naïve WT and IL5Tg mice, and in WT and IL5Tg mice injected i.v. with EO771 cells. We found that the lungs of WT mice containing EO771 tumors had an increase in the number of pulmonary eosinophils, whereas the high number of lung eosinophils in IL5Tg mice stayed relatively constant between naïve IL5Tg mice and IL5Tg mice injected with EO771 cells (**Figure 1F**; **Supplemental Figure 1B**). We found that the presence of EO771 tumors in the lungs was associated with increased levels of several pulmonary immune cell populations in WT mice, including neutrophils, alveolar macrophages, CD8^+^ T cells, and CD4^+^ T cells (**Supplemental Figure 1C**). In contrast, the majority of these immune cell populations remained relatively unaltered in the lungs of IL5Tg mice injected i.v. with EO771 cells compared to naïve IL5Tg mice, suggesting that the changes in immune cell populations in the WT mice were associated with overall tumor burden and the corresponding cytokine production and activation of the immune system. We have previously observed that mice bearing EO771 primary orthotopic tumors exhibit increased numbers of infiltrating T regulatory cells (T_regs_) in the lungs (4), which is indicative of an immunosuppressed lung microenvironment. We found that WT mice injected i.v. with EO771 cells had a 30-fold increase in the number of pulmonary T_regs_ compared to naïve WT mice, whereas T_regs_ were not elevated in the lungs of IL5Tg mice (**Figures 1G, H**). Taken together, these data suggest that EO771 tumor growth in the lungs of WT mice is associated with increased levels of several immune cell types, and that the significantly decreased EO771 growth in the lungs of IL5Tg mice is associated with the abundance of eosinophils rather than a change in the content of immune suppressive myeloid or lymphoid cells.

### Eosinophils Are Directly Cytotoxic to Tumor Cells *Ex Vivo*

**Figure 2:**
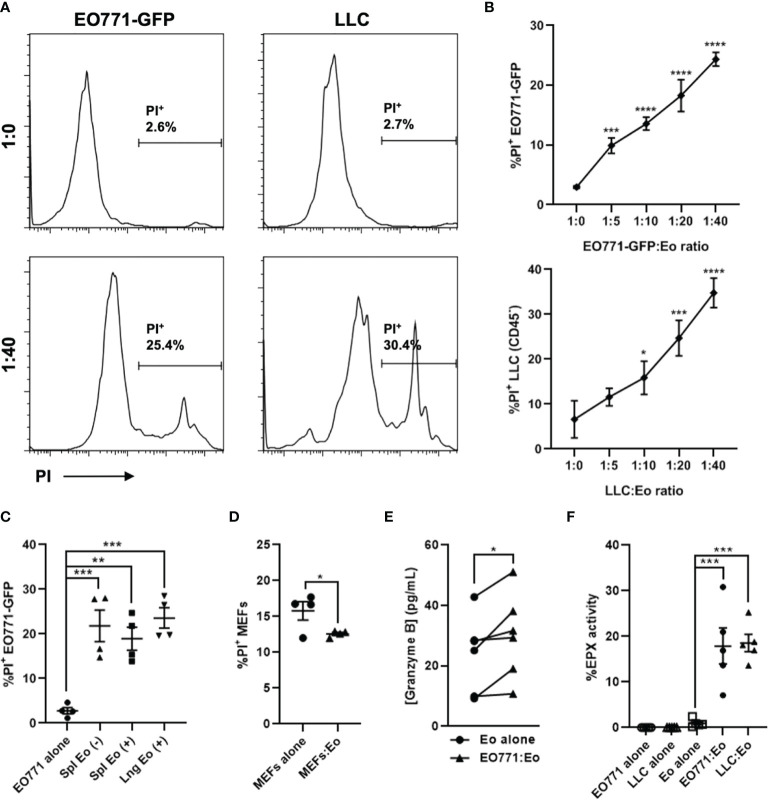
Eosinophils are directly cytotoxic to tumor cells *ex vivo*. Eosinophils (Eo) were isolated from a naïve IL5Tg spleen or lungs and co-cultured with EO771-GFP or Lewis lung carcinoma (LLC) tumor cells at varying ratios for 6 hours. **(A)** Representative histograms of PI^+^ EO771-GFP or LLC (gated on CD45^-^) tumor cells cultured alone (1:0) or co-cultured with splenic eosinophils at a 1:40 T:E ratio. **(B)** Frequency of PI^+^ EO771-GFP and LLC cells co-cultured with splenic eosinophils at indicated T:E ratios. **(C)** Frequency of PI^+^ EO771-GFP cells cultured alone or co-cultured with splenic and pulmonary eosinophils at a 1:20 T:E ratio. (+) refers to eosinophils isolated *via* positive selection and (-) refers to eosinophils isolated *via* depletion. **(D)** Frequency of PI^+^ murine embryonic fibroblasts (MEFs) cultured alone or co-cultured with splenic eosinophils at a 1:10 T:E ratio. **(E)** Quantification of Granzyme B secreted by splenic eosinophils cultured alone or with EO771-GFP cells at a 1:40 T:E ratio. **(F)** Secreted EPX activity (as measured *via* the OPD method) in supernatants of splenic eosinophil tumor cell co-cultures at a 1:40 T:E ratio, as well as eosinophils and tumor cells incubated alone. Data points are mean ± SEM of 3-6 independent experiments. *p < 0.05; **p < 0.01; ***p < 0.001; ****p < 0.0001.

We hypothesized that eosinophils were mediating the decrease in pulmonary metastatic tumor growth we observed in IL5Tg mice, and therefore we wanted to understand how eosinophils could limit tumor growth. Eosinophils have been shown to influence both the recruitment and activation of CD8^+^ T cells, although we did not observe increased CD8^+^ T cells in the lungs of naïve or EO771-injected IL5Tg mice compared to WT mice (**Supplemental Figure 1C**). Previous reports have also demonstrated that eosinophils can directly kill tumor cells *via* release of cytotoxic granule proteins (20, 23), such as MBP and EPX; therefore, we evaluated whether eosinophils could kill EO771 tumor cells *ex vivo*. We isolated spleens from naïve IL5Tg mice as a source of eosinophils and co-cultured purified eosinophils with EO771-GFP cells, as well as LLC cells, at varying T:E ratios. We found that eosinophils could kill both EO771-GFP and LLC tumor cells in a dose-dependent manner within 6 hours of co-culture (**Figures 2A, B**). We next wanted to determine if the magnitude of eosinophil-mediated tumor cell killing was dependent on the duration of the co-culture assay. Eosinophils were co-cultured with EO771-GFP tumor cells at a 1:20 T:E ratio for 6, 12, and 24 hours, and both EO771-GFP and eosinophil cell death was quantified by flow cytometry. We found that although the frequency of PI^+^ EO771-GFP cells marginally increased at 24 hours compared to 6 hours of co-culture (**Supplemental Figure 2A**), eosinophil death at both 12 hours and 24 hours in culture was significantly increased (**Supplemental Figure 2B**). Due to the high level of eosinophil death over time *in vitro*, we used a 6-hour endpoint for all subsequent co-culture experiments. To determine if the type of selection used to isolate eosinophils impacted their ability to kill tumor cells, we isolated splenic eosinophils using both depletion and positive selection in parallel and co-cultured these eosinophils with tumor cells at a 1:20 T:E ratio. We found that positive selection produced a 98% pure population of eosinophils while negative selection generated a >85% pure population of eosinophils (**Supplemental Figure 2C**) with a contaminating population of CD11b^+^Siglec-F^lo^ neutrophils. Nevertheless, we found that the selection protocol did not significantly affect tumor cell cytotoxicity (**Figure 2C**), indicating that other cells in the negatively selected eosinophil population did not influence tumor cell survival.

Due to the profound splenomegaly observed in IL5Tg mice, we used IL5Tg spleens as an abundant source of eosinophils for *ex vivo* assays. However, we wanted to confirm that pulmonary eosinophils were also able to kill tumor cells. We isolated pulmonary eosinophils from naïve IL5Tg mice (**Supplemental Figure 2C**) and co-cultured them with EO771-GFP cells at a 1:20 T:E ratio, and found that pulmonary eosinophils killed tumor cells to a similar degree as splenic eosinophils (**Figure 2C**). We also found that eosinophil-mediated cell killing was tumor cell specific, as eosinophils did not induce death of syngeneic C57Bl/6 murine embryonic fibroblasts (MEFs) in co-culture (**Figure 2D**).

Since eosinophils can secrete a multitude of factors that can induce cytotoxicity, we next collected supernatants from eosinophils co-cultured with EO771-GFP cells at a 1:40 T:E ratio and quantified TNFα, IFNγ, and Granzyme B by ELISA. While we found no detectable TNFα and IFNγ in co-culture supernatants (below level of detection, data not shown), there was a small increase in Granzyme B in co-cultures containing both eosinophils and EO771-GFP cells compared to eosinophils cultured alone (**Figure 2E**). Since Granzyme B can be a component of eosinophil granules (23, 29), these data suggest eosinophils co-cultured with EO771-GFP tumor cells may be degranulating. To generate an additional measure of eosinophil degranulation, we collected supernatants from eosinophil-tumor cell co-cultures at 1:10, 1:20, and 1:40 T:E ratios and assessed peroxidase activity *via* the OPD method. We found that there was an increase in peroxidase activity when eosinophils were co-cultured with either EO771-GFP or LLC cells at a 1:40 T:E ratio compared to eosinophils or tumor cells cultured alone (**Figure 2F**). These data suggest that the presence of tumor cells causes eosinophils to release peroxidase, which is normally contained within eosinophil intracellular granules. Similarly, we found an increase in peroxidase activity in co-cultures containing eosinophils and EO771-GFP tumor cells at both 1:10 and 1:20 T:E ratios compared to eosinophils cultured alone (**Supplemental Figure 2D**). Though the OPD method has been widely utilized to quantify eosinophil peroxidase (EPX) activity (25, 26), the assay detects total peroxidase activity as opposed to specific EPX activity. While EPX is the only peroxidase present in eosinophil granules, we wanted to confirm that peroxidase activity derived from co-cultures of tumor cells and negatively selected eosinophils was not potentially due to neutrophil-derived myeloperoxidase. We isolated eosinophils *via* positive and negative selection as above and co-cultured them with EO771-GFP cells at a 1:40 T:E ratio. We found that co-cultures containing positively selected (98% pure) eosinophils had higher peroxidase activity compared to co-cultures containing negatively selected eosinophils from the same mouse (**Supplemental Figure 2E**). These data indicate that the peroxidase activity in the tumor cell:eosinophil co-cultures is eosinophil-derived and therefore represents EPX released by eosinophil degranulation *in vitro*. Taken together, these data indicate that EO771-GFP and LLC cells can induce eosinophil degranulation and are sensitive to eosinophil-mediated cytotoxicity *in vitro*, thus identifying direct tumor cell killing as a potential mechanism by which eosinophils can reduce EO771 metastatic growth *in vivo*.

### Eosinophils Inhibit Early Pulmonary Colonization of EO771 Tumor Cells in IL5Tg Mice

**Figure 3:**
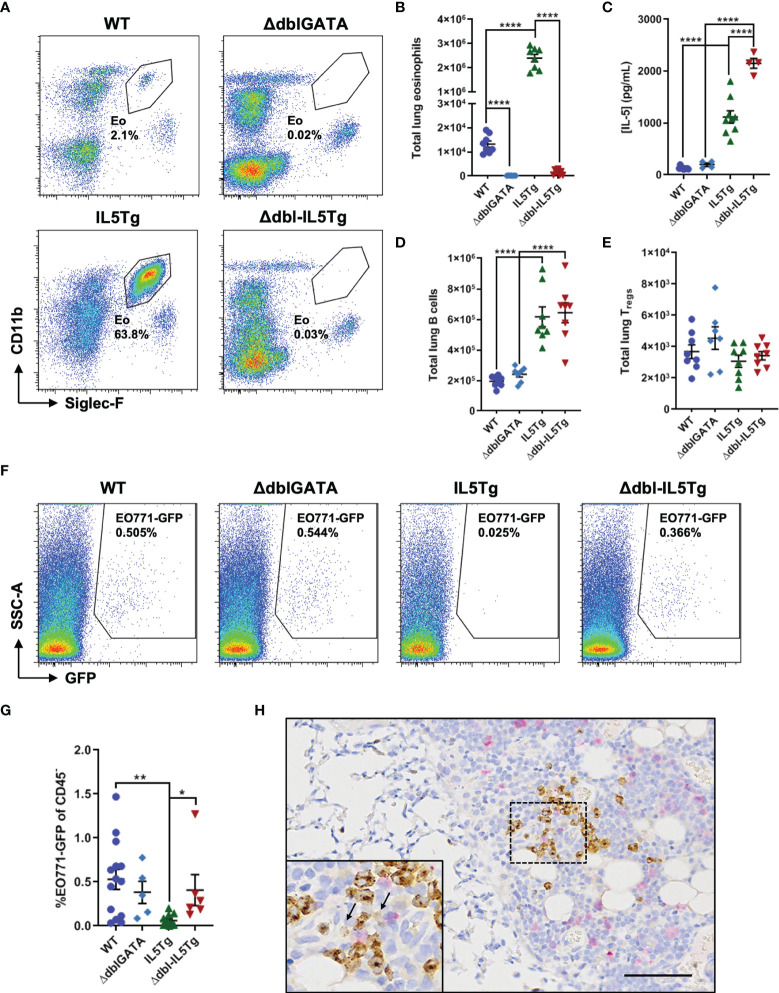
Eosinophils inhibit early pulmonary colonization of EO771 tumor cells in IL5Tg mice. **(A)** Representative flow plots of lungs collected from naïve WT, ΔdblGATA, IL5Tg, and ΔdblGATA-IL5Tg (Δdbl-IL5Tg) mice. **(B)** Total number of eosinophils in the left lung lobe of naïve WT, IL5Tg, ΔdblGATA, and Δdbl-IL5Tg mice. **(C)** Quantification of circulating IL-5 in the serum of WT, ΔdblGATA, IL5Tg, and Δdbl-IL5Tg mice. **(D)** Total number of B cells (CD19^+^) and **(E)** T_regs_ in the left lung lobe of naïve WT, IL5Tg, ΔdblGATA, and Δdbl-IL5Tg mice. **(F)** Representative flow plots of WT, ΔdblGATA, IL5Tg, and Δdbl-IL5Tg mouse lungs 5 days post-i.v. injection of EO771-GFP cells. Samples pre-gated on CD45^-^ cells. **(G)** Frequency (as a proportion of CD45^-^ cells) of EO771-GFP cells in the lungs of WT, ΔdblGATA, IL5Tg, and Δdbl-IL5Tg mice 5 days post-i.v. injection of EO771-GFP cells. **(H)** Representative lung IHC of an IL5Tg mouse 6 hours post-i.v. injection of EO771-GFP cells. Sections were co-stained with anti-mMBP to identify eosinophils (brown) and anti-GFP to visualize EO771 tumor cells (pink). Black arrows in inset indicate diffuse MBP staining indicative of degranulating eosinophils. Scale bar = 100μm. Data points are individual mice with mean ± SEM of 5-14 mice per group. *p < 0.05; **p < 0.01; ****p < 0.0001.

Though IL5Tg mice have been previously used to study the specific role of eosinophils in a number of disease states (11), their profound IL-5 overexpression can result in additional changes within the immune compartment in different tissues and systemically. In order to specifically examine the role of eosinophils in the pulmonary metastatic microenvironment, we used eosinophil-deficient ΔdblGATA mice and ΔdblGATA/IL5Tg (Δdbl-IL5Tg) double transgenic mice. ΔdblGATA mice have a deletion within the high-affinity GATA-binding site located in the eosinophil specific promoter region of the GATA-1 transcription factor, resulting in an early block in eosinophil development while sparing red cell, platelet and mast cell development (30). As a further control for any potential eosinophil-independent impact of IL-5 overexpression on the lung microenvironment, we also crossed ΔdblGATA mice with IL5Tg mice to generate Δdbl-IL5Tg mice, which have elevated IL-5 expression in the absence of eosinophils (**Figures 3A–C**).

We used flow cytometry to quantify potential changes to the pulmonary immune microenvironment across all four genotypes and found a significant increase in the number of lung B cells in IL5Tg mice relative to WT mice (**Figure 3D**), as well as increased B cells in the blood and spleen (data not shown). These alterations within the B cell compartment have been previously demonstrated in IL5Tg mice (28). Importantly, we found that eosinophil deficient Δdbl-IL5Tg mice have similarly elevated levels of pulmonary B cells as IL5Tg mice (**Figure 3D**), which will allow us to distinguish between eosinophil-associated and potential B cell-associated changes in EO771 growth in the lungs. We found no significant differences in the total number of pulmonary T_regs_ (**Figure 3E**), CD4^+^ T cells, or alveolar macrophages across all four genotypes, with a small increase in lung neutrophils detected in naïve IL5Tg mice compared to WT mice (**Supplemental Figure 3**). Additionally, ΔdblGATA mice had a trend towards an increase in the number of pulmonary CD8^+^ T cells compared to WT mice, though this did not reach statistical significance (**Supplemental Figure 3**). Since the most striking changes within the lung immune compartment across all four genotypes were eosinophils, these transgenic mouse models allowed us to delineate the specific role of eosinophils in pulmonary metastatic colonization and growth.

We were first interested in whether eosinophils could prevent early *in vivo* pulmonary metastatic colonization. In order to test if eosinophils could inhibit the initial pulmonary seeding of EO771 tumor cells, we injected WT, ΔdblGATA, IL5Tg, and Δdbl-IL5Tg mice i.v. with EO771-GFP cells and quantified the frequency of GFP^+^ tumor cells in the lungs 5 days later. We found that IL5Tg mice had a significant decrease in the frequency of EO771-GFP tumor cells 5 days post-i.v. injection compared to WT and Δdbl-IL5Tg mice (**Figures 3F, G**), indicating that eosinophilia (and not increased B cells) is associated with a decrease in early tumor cell colonization of the lungs in IL5Tg mice. Subsequent staining of lung sections with antibodies against mMBP and GFP allowed us to visualize MBP^+^ eosinophils (**Supplemental Figure 4A**) and GFP^+^ tumor cells (**Supplemental Figure 4B**). We found clusters of GFP^+^ tumor cells in the lungs of WT mice, whereas we detected rare single tumor cells scattered throughout the lungs of IL5Tg mice 5 days after injection (**Supplemental Figure 4B**). However, there was no difference in EO771-GFP lung colonization between WT and eosinophil-deficient ΔdblGATA or Δdbl-IL5Tg mice 5 days after i.v. injection (**Figures 3F, G**), indicating that tumor cell colonization is not impaired by eosinophils in the lungs of WT mice. We next wanted to determine if eosinophils in the lungs of IL5Tg mice were degranulating in proximity to EO771-GFP tumor cells. Since we observed eosinophil degranulation and eosinophil-mediated tumor cell killing within 6 hours of co-culture *ex vivo* (**Figure 2**), we stained lungs from IL5Tg mice with anti-mMBP and anti-GFP 6 hours after i.v. injection of EO771-GFP cells. We found substantial numbers of tumor cells in the pleura and mediastinum (**Figure 3H**; pink), as well as single GFP^+^ tumor cells scattered throughout the lung parenchyma (**Supplemental Figure 4C**). When MBP^+^ eosinophils were observed near GFP^+^ tumor cells in the pleura, we noted areas of diffuse extracellular MBP staining indicative of eosinophil degranulation (**Figure 3H**). Taken together, our data indicate that eosinophils can degranulate in close proximity to tumor cells in the lung, that eosinophil degranulation occurs rapidly near tumor cells, and that eosinophilia is associated with a decrease in early lung colonization by i.v. injected EO771 tumor cells.

### Pulmonary Eosinophils Become Activated During EO771 Tumor Growth in the Lungs

**Figure 4:**
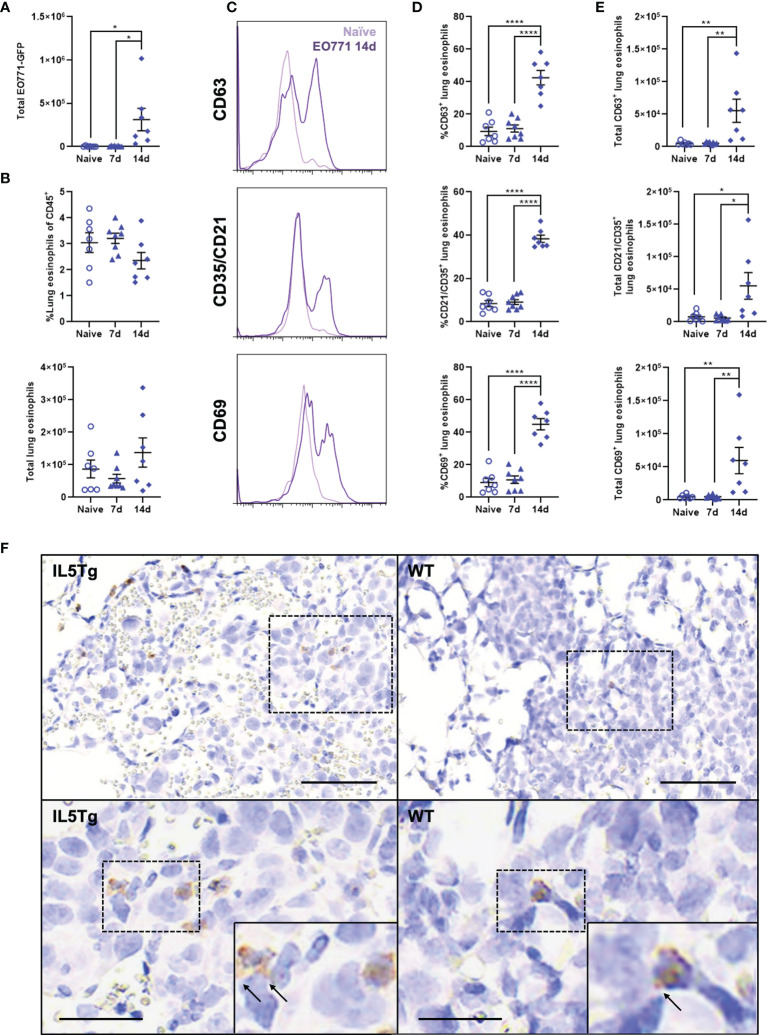
Lung eosinophils become activated during pulmonary EO771 tumor growth. **(A)** Total number of EO771-GFP cells in the lungs of WT mice 7 days and 14 days following i.v. injection of EO771-GFP cells. **(B)** Frequency and total number of lung eosinophils in naïve WT mice and WT mice injected i.v. with EO771-GFP cells 7 days and 14 days post-injection. **(C)** Representative histogram overlays of eosinophil expression of CD63, CD35/CD21, and CD69 in naïve WT mice and WT mice 14 days after i.v. injection with EO771-GFP cells. **(D)** Frequency and **(E)** total number of eosinophils expressing CD63, CD35/CD21, and CD69 in naïve WT mice and mice either 7 days or 14 days post-i.v. injection of EO771-GFP cells. **(F)** Representative lung IHC of WT and IL5Tg mice 14 days after i.v. injection of EO771-GFP cells. Sections were stained with anti-mMBP and anti-GFP to visualize eosinophils (brown) and EO771-GFP tumor cells (pink). Black arrows indicate extracellular MBP staining. Scale bar = 100μm (upper images) and 50μm (lower images). Data points are individual mice with mean ± SEM of 7-8 mice per group. *p < 0.05; **p < 0.01; ****p < 0.0001.

Eosinophils isolated from IL5Tg mice degranulate *ex vivo* when co-cultured with EO771 tumor cells (**Figure 2**), and eosinophils in IL5Tg mice can degranulate near EO771 tumor cells *in vivo* and restrict lung colonization (**Figures 3F–H**). However, we observed no difference in pulmonary colonization of EO771-GFP cells in WT and eosinophil-deficient ΔdblGATA 5 days post-i.v. injection, suggesting that eosinophils do not inhibit early metastatic colonization in WT mice. Eosinophils are present in relatively low numbers in naïve WT mice compared to IL5Tg mice (**Figure 3B**), so it is perhaps not surprising that lung colonization was not significantly impacted by eosinophils in WT mice. We previously noted increased numbers of eosinophils in the lungs of WT mice 3 weeks after EO771 tumor cell injection (**Figure 1F**), and were curious about the number and activation status of eosinophils during EO771 tumor growth in the lungs. We injected WT mice with EO771-GFP cells and harvested lungs at 7 days and 14 days post-i.v. injection. As expected, the total number of pulmonary EO771-GFP cells increased 14 days post-i.v. injection (**Figure 4A**) as tumor nodules grew in the lungs, although the frequency and total number of eosinophils did not significantly change over this time period (**Figure 4B**).

A population of Siglec-F^hi^ eosinophils were recently shown to be recruited to the lungs following breast cancer lung metastasis (31), and we wanted to determine if EO771 tumors induce changes in the fraction of resident or recruited eosinophils in the lungs during tumor growth. We found that there was no change in the total number of Siglec-F^mid^ lung-resident eosinophils, whereas there was a small increase in the total number of recruited Siglec-F^hi^ eosinophils 14 days post-i.v. injection of EO771-GFP cells (**Supplemental Figure 5A, B**). This resulted in a significant decrease in the proportion of Siglec-F^mid^ lung-resident eosinophils and concomitant increase in the proportion of Siglec-F^hi^ recruited eosinophils (**Supplemental Figures 5C, D**). We also quantified expression of CD63, CD35/CD21, and CD69 on eosinophils during EO771 tumor growth in the lungs since these are established markers of eosinophil activation and/or secretion (32, 33). Interestingly, we found that although eosinophil expression of CD63, CD35/CD21, and CD69 was similar between naïve WT mice and WT mice 7 days following EO771-GFP i.v. injection, both the frequency (**Figures 4C, D**) and total number of activated eosinophils (**Figure 4E**) were significantly higher 14 days following EO771-GFP i.v. injection. These data indicate that eosinophils in the lungs of WT mice become activated and express markers associated with degranulation during the growth of EO771 tumors in the lungs. Lastly, we stained lung sections from WT and IL5Tg mice at 14 days post-i.v. injection of EO771-GFP cells with anti-mMBP and anti-GFP to visualize eosinophils and tumor cells, respectively. In line with our previous flow cytometric analysis (**Supplemental Figure 6B**), we identified numerous MBP^+^ eosinophils located in IL5Tg lung tumor nodules, and few eosinophils in WT lung tumor nodules (**Figure 4F**). We detected both diffuse and extracellular MBP staining adjacent to MBP^+^ eosinophils, indicative of eosinophil degranulation within tumor nodules in both WT and IL5Tg mice (**Figure 4F**). In combination, these data indicate that eosinophils become activated and more secretory as EO771 lung tumors progress.

### Genetic or Immunological Eosinophil Depletion Accelerates Pulmonary EO771 Tumor Growth

**Figure 5:**
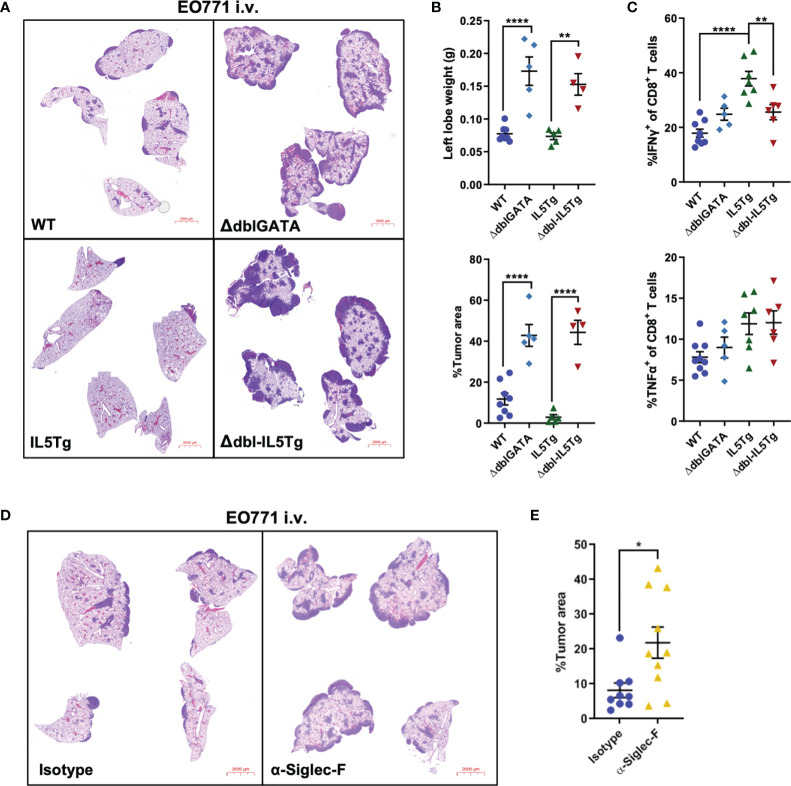
Genetic or immunological eosinophil depletion accelerates pulmonary EO771 tumor growth. **(A)** Representative images of lungs harvested from WT, IL5Tg, ΔdblGATA, and Δdbl-IL5Tg mice injected i.v. with EO771 cells. Lungs were harvested 14 days post-injection due to accelerated growth in eosinophil-deficient ΔdblGATA and Δdbl-IL5Tg mice. **(B)** WT, IL5Tg, ΔdblGATA, and Δdbl-IL5Tg left lung lobe weights and EO771 tumor area quantification as a proportion of total lung area of right lung lobes. Data is from one independent experiment, which has been replicated using EO771-GFP cells. **(C)** Frequency of IFNγ^+^ and TNFα^+^ *ex vivo* stimulated CD8^+^ lung T cells harvested from WT, IL5Tg, ΔdblGATA, and Δdbl-IL5Tg mice injected i.v. with EO771 cells. **(D)** Representative images of lungs from WT mice injected i.v. with EO771 cells and treated the next day with 0.6mg/kg α-Siglec-F or IgG_2a_ isotype control and every 5 days thereafter. Lungs were harvested 13 days post-i.v. EO771 injection. **(E)** Quantification of EO771 tumor area as a proportion of total lung area of right lung lobes. Data points are individual mice with mean ± SEM of 4-10 mice per group. *p < 0.05; **p < 0.01; ****p < 0.0001.

Though eosinophils in WT mice did not impact early EO771-GFP metastatic colonization (**Figure 3G**), we were interested in whether WT eosinophils affect subsequent growth of EO771 tumors in the lungs. To assess the impact of eosinophil deficiency on EO771 pulmonary metastatic growth, we injected WT, IL5Tg, ΔdblGATA, and Δdbl-IL5Tg mice i.v. with EO771 cells and allowed lung nodules to form over time. Strikingly, both eosinophil-deficient mouse strains (ΔdblGATA and Δdbl-IL5Tg) displayed an accelerated disease phenotype and reached humane endpoint 14 days post-i.v. injection (as opposed to the 21-day experimental endpoint used for WT mice in **Figure 1**). We found that both ΔdblGATA and Δdbl-IL5Tg mice had a large increase in EO771 tumor burden when compared to WT and IL5Tg mice, respectively, which is demonstrated by both an increase in lung weight and lung tumor area determined histologically (**Figures 5A, B**). Our findings indicate that EO771 lung colonization is decreased in eosinophilic IL5Tg mice, and that pulmonary EO771 tumor growth is increased in eosinophil-deficient ΔdblGATA and Δdbl-IL5Tg mice.

As we had previously demonstrated that eosinophils in WT mice upregulate markers of activation (CD69, CD35/CD21) and secretion (CD63) 14 days after EO771 tumor cell injection (**Figure 4**), we were interested in whether pulmonary eosinophils in IL5Tg lungs are similarly activated after i.v. EO771-GFP injection. We found that the lungs of naïve tumor-free IL5Tg and WT mice contain small proportions of activated eosinophils, although the eosinophilia in IL5Tg mice is associated with a higher number of total activated eosinophils relative to WT mice (**Supplemental Figures 7A, B**). The number of activated and degranulating eosinophils in the lungs of WT mice increases 2 weeks after i.v. injection (**Figure 4**), and we observed comparably high levels of CD35/CD21^+^ and CD69^+^ activated eosinophils in IL5Tg mice, with a greater abundance of degranulating CD63^+^ eosinophils in IL5Tg mice 2 weeks after tumor cell injection (**Supplemental Figure 7C**). These data indicate that 14 days after tumor cell injection, the lungs of WT mice contain increased numbers of activated degranulating eosinophils that are comparable to the numbers of activated pulmonary eosinophils found in IL5Tg mice.

We were interested in the lung immune cell compartment in WT, IL5Tg, ΔdblGATA, and Δdbl-IL5Tg mice 14 days after EO771 injection. Consistent with the increased tumor burden observed in ΔdblGATA and Δdbl-IL5Tg mice, we found several different immune cell populations were elevated in the lungs of these mice 2 weeks after tumor cell injection compared to naïve tumor-free mice, including neutrophils, alveolar macrophages, CD8^+^ T cells, CD4^+^ T cells, and T_regs_, whereas there was no significant difference at this timepoint between naïve and EO771-bearing WT or IL5Tg mice (**Supplemental Figure 6**). These data are similar to our observations of elevated immune cell populations in WT mice with high tumor burden 21 days after EO771 tumor cell injection (**Supplemental Figure 1B**), suggesting recruitment of these immune cells to the lungs is associated with the high tumor burden and not the lack of eosinophils in these models. Overall, our data show that the lack of eosinophils in ΔdblGATA and Δdbl-IL5Tg mice leads to increased EO771 tumor growth in the lungs over time compared to WT mice.

Eosinophils were previously shown to enhance T cell-mediated anti-tumor immunity in a mouse model of breast cancer metastasis (31). As such, we were interested in T cell cytokine production in the lungs of eosinophil-deficient and eosinophilic mice during EO771-GFP lung tumor growth. WT, IL5Tg, ΔdblGATA, and Δdbl-IL5Tg mice were injected i.v. with EO771-GFP cells and lungs were harvested 14 days later to assess T cell cytokine production *ex vivo*. We found that there was no difference in TNFα production in both the CD8 and CD4 T cell compartment across all four genotypes (**Figure 5C** and **Supplemental Figures 8A, B**). Interestingly, we found a significant increase in IFNγ production in both CD8^+^ and CD4^+^ T cells in the lungs of IL5Tg mice compared to both WT and Δdbl-IL5Tg mice (**Figure 5C** and **Supplemental Figure 8B**), suggesting that eosinophilia and reduced tumor burden in IL5Tg mice correlates with increased proportions of IFNγ producing T cells. Although the proportion of IFNγ^+^ CD4 T cells was significantly lower in Δdbl-IL5Tg mice compared to IL5Tg mice, it was significantly higher compared to ΔdblGATA mice, suggesting an eosinophil-independent role for IL-5 in CD4 T cell production of IFNγ (**Supplemental Figure 8B**). However, we found there was no difference in T cell IFNγ production between WT and ΔdblGATA mice (**Figure 5C**; **Supplemental Figure 8B**). These data demonstrate that the increased EO771 tumor growth in the absence of eosinophils in ΔdblGATA mice is not due to reduced proportions of IFNγ or TNFα producing T cells. These data further support a direct role of eosinophils in WT mice in reducing pulmonary tumor burden relative to eosinophil deficient mice.

Lastly, we employed antibody-mediated depletion of eosinophils in WT mice to validate our previous findings in ΔdblGATA and Δdbl-IL5Tg mice. WT mice were injected i.v. with EO771 tumor cells and treated with anti-Siglec-F to deplete eosinophils (or IgG_2a_ isotype control), and lungs were harvested 13 days post-injection. Since *in vivo* treatment with anti-Siglec-F can render flow cytometry-based identification of Siglec-F^+^ cells ineffective, we utilized an alternative gating strategy to identify eosinophils and confirm eosinophil depletion (**Supplemental Figure 9A**). As previously shown (34), anti-Siglec-F depleted lung eosinophils while mostly sparing Siglec-F^+^ alveolar macrophages (**Supplemental Figures 9B, C**). We found that antibody-mediated depletion of eosinophils in WT mice resulted in a significant increase in EO771 lung tumor burden (**Figure 5D, E**), which is consistent with data from our eosinophil deficient genetically-engineered mouse models and confirms that eosinophils decrease EO771 tumor growth in the lungs. Interestingly, we observed increased CD8^+^ T cells in the lungs of mice treated with anti-Siglec-F compared to isotype-treated mice, while we did not find any significant differences in CD4^+^ T cells, T_regs_, B cells, or neutrophils (**Supplemental Figure 10**). These data further support that the influence of eosinophils on inhibiting metastatic growth of EO771 cells is not driven through indirect modification of the T cell compartment. In summary, our data indicate that eosinophils can directly kill EO771 tumor cells *in vitro*, that eosinophils become activated following pulmonary metastatic colonization, and that EO771 tumor nodules in the lungs grow more rapidly in the absence of eosinophils.

## Discussion

In this study, we investigated the role of eosinophils in pulmonary metastatic mammary tumor growth. We found that IL5Tg mice with elevated lung eosinophils have a marked decrease in pulmonary colonization by EO771 mammary carcinoma cells compared to WT mice. Though eosinophils in naïve WT lungs comprise only ~3% of total pulmonary leukocytes, WT mice have a significant reduction in metastatic tumor growth in the lungs compared to eosinophil-deficient ΔdblGATA and Δdbl-IL5Tg mice, as well as WT mice treated with anti-Siglec-F to deplete eosinophils. Further, eosinophils directly kill tumor cells *ex vivo* and can degranulate in proximity to tumor cells *in vivo*. These data highlight a role for eosinophils in the control of pulmonary metastatic tumor growth.

Transgenic mouse models of eosinophilia and eosinophil-deficiency, as well as antibody-mediated eosinophil depletion, have been previously utilized to study the role of eosinophils in various models of disease (11). Recently, the role of these widely ignored immune cells in the tumor microenvironment has gained additional attention, in part due to the growing accrual of data indicating that eosinophils accumulate in both the primary tumor, circulation, and metastases of cancer patients (12–15, 35–37), as well as pre-clinical studies demonstrating both the pro- and anti-tumorigenic role of eosinophils (7, 8, 17, 20–22). While systemic IL-5 production in IL5Tg mice drives the accumulation of eosinophils in the lungs, previous studies have shown that cancer cells and host cells can produce factors which recruit eosinophils to the tumor microenvironment, such as IL-5, CCL11, and various damage-associated molecular patterns (DAMPs). Recently, CCL11 expression detected in gastrointestinal punch biopsies of colorectal cancer (CRC) patients was shown to correlate with eosinophil infiltration into tumor nodules (21). Similar to our findings, the study also demonstrated that eosinophil-deficient ΔdblGATA mice had decreased survival in a mouse model of CRC, highlighting a similar anti-tumorigenic role for eosinophils in gastrointestinal malignancies (21). The alarmin IL-33, which stimulates the production of IL-5 from group 2 innate lymphoid cells (ILC2s) and results in eosinophil recruitment, has also been studied in the context of peritoneal and pulmonary metastasis. The induction of Th2-driven airway inflammation with either IL-33 or *Aspergillus* protease allergen was recently shown to increase experimental lung metastasis by B16 melanoma and spontaneous lung metastasis by 4T1 mammary carcinoma cells (18). In contrast, treatment of mice with IL-33 has also been shown to decrease metastasis in murine models of ID8 ovarian cancer peritoneal metastasis (38) and B16 melanoma pulmonary metastasis (22). Likewise, IL-33 and anti-PD-1 combination therapy was recently shown to unbridle anti-tumor immunity mediated by an ILC2-eosinophil axis in a murine model of melanoma (39). Though differences in IL-33 dosing, and the timing of said dosing, may explain the conflicting role of eosinophils in these reports, eosinophils can also be influenced by the local and distal microbiota. For instance, germ-free (GF) mice were recently shown to have more eosinophils at mucosal sites; however, their ability to degranulate was impaired (40). While microbiota differences may contribute to these conflicting pre-clinical results, the role of the microbiome in dictating eosinophil phenotype within the metastatic microenvironment has yet to be characterized.

Emerging data also suggest that eosinophil phenotype and effector function can be influenced by the local microenvironment. In our study, we used an established experimental model of lung colonization to study the influence of eosinophils on tumor cell colonization and growth in the lungs. While our current study does not address the effect of the primary tumor on the formation of distal, pre-metastatic niches, previous work from our lab has shown that breast cancer-secreted factors can foster an immune-suppressive lung microenvironment (2–4). Further studies are required to assess the impact of the primary tumor on lung-resident eosinophils, recruited eosinophils, and spontaneous breast cancer pulmonary metastasis. However, studies which explore the role of specific microenvironmental factors on eosinophil function have demonstrated that eosinophil phenotype is varied in the context of cancer (17, 20–22). Similar to neutrophils and macrophages, eosinophils are likely a heterogenous population of innate immune cells, with a propensity towards either a pro- or anti-tumor phenotype depending on the local microenvironment. Though this remains to be thoroughly investigated in the context of cancer, two distinct eosinophil populations (Siglec-F^mid^ and Siglec-F^hi^) with opposing effector functions have been identified in the lungs of allergen-challenged mice (41), and the Siglec-F^hi^ subpopulation was recently shown to accumulate in the lungs of mice harboring metastatic breast cancer (31). We observed a small increase in recruited Siglec-F^hi^ eosinophils during EO771 mammary tumor growth, though to a lesser degree than other mammary tumor models. This potential plasticity or multiple cellular subsets may explain conflicting roles of eosinophils in both pre-clinical models and human cancer.

There have been various mechanisms proposed by which eosinophils limit tumor cell growth or metastatic colonization, including modulating tumor immune infiltration, vascular normalization, and direct tumor cell killing. Eosinophils can be easily detected *via* eosin staining due to the cationic effector proteins located in their granules, which include MBP, EPX, ECP, and EDN (42). Though the biochemical effector functions differ between these granule proteins, in general, they are directly cytotoxic to a number of bacteria, viruses, and parasites, and this process of cytotoxic degranulation is one of the primary functions of eosinophils (6). However, EDN has also been shown to signal through Toll-like receptor 2 (TLR2) expressed by dendritic cells, highlighting an additional potential mechanism for enhanced anti-tumor immunity (43). Recent pre-clinical studies have demonstrated that eosinophils can directly kill a variety of tumor cell types, including CRC, melanoma, hepatocellular carcinoma, and breast cancer (20–22). We have found that eosinophils isolated from IL5Tg mice can kill both EO771 and LLC tumor cells *ex vivo*. Using our co-culture system, we demonstrated that EPX is released upon co-culture of eosinophils with tumor cells, suggesting that eosinophil degranulation may be a potential mechanism by which eosinophils can kill tumor cells. We also found evidence of degranulation *in vivo* following i.v. injection of EO771-GFP cells, as eosinophils in WT mice upregulate activation and degranulation markers 14 days after EO771-GFP injection, and eosinophils in IL5Tg mice rapidly degranulate in close proximity to tumor cells in the lung pleura and parenchyma. However, eosinophils are also known to produce several additional factors that can have cytotoxic activity, including granzymes A and B, TNFα, and IFNγ. We found that eosinophils co-cultured *in vitro* with EO771-GFP cells marginally increase secretion of Granzyme B, which is released *via* degranulation, and that there were no detectable TNFα or IFNγ in culture supernatants. Interestingly, *ex vivo* eosinophil-mediated tumor cell killing *via* degranulation was recently shown to be partially dependent on CD11b/CD18-mediated adhesion of eosinophils to tumor cells (23), suggesting that physical contact between eosinophils and tumor cells is necessary for eosinophil-mediated killing. We found that eosinophils do not kill MEFs, indicating that eosinophils can distinguish normal cells from malignant cells. Preliminary studies indicate that eosinophils may recognize tumor cells *via* interactions through cell surface 2B4 (44) and NKG2D (45), and indeed we find that eosinophils isolated from IL5Tg mice express both cell surface molecules (data not shown). However, the specific mechanism by which eosinophils recognize malignant EO771 cells remains unclear.

Apart from being directly cytotoxic to tumor cells, eosinophils can also indirectly modulate the tumor microenvironment through the production of chemokines and cytokines. Eosinophils were recently shown to recruit CCR1^+^ B16 melanoma cells to the lungs and bone marrow *via* the secretion of CCL6 (16). In contrast to our findings, the authors found an increase in melanoma bone marrow metastasis utilizing a similar transgenic mouse model of IL-5-driven eosinophilia. These conflicting reports may be due to differences between pulmonary and bone marrow metastatic microenvironments, or tumor cell-intrinsic factors, such as differences in CCR1 expression. Similarly, eosinophils have also been reported to facilitate LLC pulmonary metastatic colonization through CCL22-mediated recruitment of T_regs_ to the lungs (17), although our *ex vivo* data indicate LLC cells can be directly killed by eosinophils. In this study, the authors relied primarily on IL5-KO mice to study the role of eosinophils in pulmonary metastasis. Though IL5-KO mice have a reduction of pulmonary eosinophils, eosinophils still develop due to the compensatory effects of IL-3 and GM-CSF (46). Additionally, the absence of IL-5 results in deficits within the B-1 B cell compartment, as well as the development of IgA^+^ plasma cells (46, 47). Using IL5Tg and Δdbl-IL5Tg mice in our study allowed us to directly discriminate between the influence of IL-5 on eosinophils and B cells, allowing us to conclude that eosinophils drive the IL-5-mediated decrease in EO771 pulmonary tumor burden. However, the usage of ΔdblGATA mice is not without caveats; ΔdblGATA mice harbor deficits within the basophil compartment (48), and basophils have recently been shown to recruit CD8^+^ T cells in models of murine melanoma (49). We used anti-Siglec-F to specifically deplete eosinophils (and not basophils) in WT mice to confirm a role for eosinophils in limiting pulmonary metastatic growth, although further experiments are necessary to investigate the potential anti-tumorigenic role of basophils in ΔdblGATA mice. Conversely, eosinophils have been shown to produce chemokines, such as CCL5, CXCL16, CXCL9, and CXCL10, that actively recruit CD4^+^ and CD8^+^ T cells to the primary tumor in a murine model of melanoma (8) and breast cancer pulmonary metastases (31), as well as promote skewing of CD4^+^ and CD8^+^ T cells towards an anti-tumorigenic Th1 phenotype in CRC (50). However, we found that there was no difference in IFNγ or TNFα production in either CD4^+^ or CD8^+^ T cell populations in eosinophil-deficient ΔdblGATA mice compared to WT mice. These data indicate that eosinophil-mediated alterations to the T cell compartment are likely not contributing to the increased tumor burden we observed in eosinophil-deficient mice. Interestingly, accumulation of eosinophils in the tumor microenvironment also correlates with vascular normalization, in both murine models of melanoma (8) and breast cancer (7), which may allow for improved immune cell infiltration and intratumoral drug delivery. Indeed, increased peripheral blood eosinophils and eosinophil infiltration into murine breast carcinoma tumors was shown to be correlated with improved response to anti-CTLA4 therapy, as well as vessel normalization (7), suggesting a potential indirect anti-tumorigenic role for eosinophils in the tumor microenvironment.

Similar to pre-clinical studies, patient data also indicates a heterogenous role for eosinophils in various cancer types, which has been extensively reviewed elsewhere (51). For example, eosinophils seem to play an anti-tumorigenic role in CRC (12, 13) and breast cancer (52, 53). Breast cancer patients with a higher baseline blood eosinophil count had improved disease-free survival compared to patients with lower eosinophil blood counts (53). In contrast, increased tissue eosinophilia in patients diagnosed with cervical cancer (14, 15) or Hodgkin’s lymphoma (54) predicted worse overall survival, and increased tumor invasion in the case of cervical cancer, demonstrating that the role of eosinophils in cancer progression is likely dependent on the specific tissue microenvironment. While pre-clinical and patient studies have identified a significant role for eosinophils in the tumor microenvironment, their role in patient treatment response remains largely unstudied. Interestingly, increased peripheral blood eosinophil counts in patients receiving checkpoint blockade therapy for metastatic melanoma (35, 36) and NSCLC (55, 56) has been associated with therapy response. It remains to be determined whether eosinophils are directly involved in immunotherapy response, or whether the correlative increase in blood eosinophils is a bystander effect of improved anti-tumor immunity. Various clinical monoclonal antibodies targeting either IL-5 or the IL-5R have been developed for the treatment of eosinophilic asthma (57), but therapeutics which recruit or activate eosinophils are lacking. However, a recent study identified a small molecule inhibitor of dipeptidyl peptidase DPP4 (iDPP4) which resulted in an eosinophil-dependent decrease in tumor growth, in preclinical models of hepatocellular carcinoma and breast cancer (20). Additionally, combining DPP4i treatment with immune checkpoint blockade caused tumor regression (20). Though still in the pre-clinical stage of development, these data demonstrate both the feasibility of designing therapeutics to target eosinophils and the potential improved efficacy of combining such therapeutics with existing checkpoint blockade therapies. Further studies are necessary to characterize local and distal factors which influence eosinophil effector functions. Identifying factors which influence eosinophil phenotype in various cancer types will be critical for selecting patients who may respond to novel eosinophil-targeted therapeutics.

Immunotherapies that harness the cytotoxic potential of CD8^+^ T cells have revolutionized the treatment of metastatic disease. Despite this, many patients do not respond to existing immunotherapies, emphasizing the need to identify new immune cell subsets with the potential to kill cancer cells or enhance the cytotoxic effector functions of T cells and/or NK cells. We demonstrate here that eosinophils decrease pulmonary metastatic growth of EO771 mammary tumor cells. Following metastatic tumor cell colonization, pulmonary eosinophils upregulate markers of activation and degranulation. Eosinophils can directly kill tumor cells *in vitro*, demonstrating that eosinophils may play an important role as cytotoxic effector cells of anti-tumor immunity. Our data provide support for developing therapeutics that exploit the tumor cell cytotoxic activity of eosinophils as a potential therapeutic strategy.

## Data Availability Statement

The raw data supporting the conclusions of this article will be made available by the authors, without undue reservation.

## Ethics Statement

The animal study was reviewed and approved by the Canadian Council on Animal Care and the University of British Columbia Animal Care Committee.

## Author Contributions

RC conducted the majority of the experiments, analyzed and interpreted most of the data, and wrote the manuscript. SF, BW, AS, LD, MH, and RS assisted with tissue collection and image analysis. MRH assisted with the animal breeding colony. KM and KB contributed intellectually to study design, data interpretation, and manuscript revision. KB supervised the study. All authors read and approved the manuscript.

## Funding

This work was funded by the Cancer Research Society (24042) and the Canadian Institutes of Health Research (CIHR; PJT#159513). RC, BW, and RS were funded by CIHR Doctoral Research Awards; MH was funded by a CIHR Canada Graduate Scholarship.

## Conflict of Interest

The authors declare that the research was conducted in the absence of any commercial or financial relationships that could be construed as a potential conflict of interest.

## Publisher’s Note

All claims expressed in this article are solely those of the authors and do not necessarily represent those of their affiliated organizations, or those of the publisher, the editors and the reviewers. Any product that may be evaluated in this article, or claim that may be made by its manufacturer, is not guaranteed or endorsed by the publisher.
