# Supplementary material for: Eosinophils Decrease Pulmonary Metastatic Mammary Tumor Growth
Source: Front Oncol. 2022 Jun 8;12:841921. doi: 10.3389/fonc.2022.841921 (PMC9213661; doi:10.3389/fonc.2022.841921)
Supplement: Supplementary file 1 [file Presentation_1.pptx]

## Slide 1
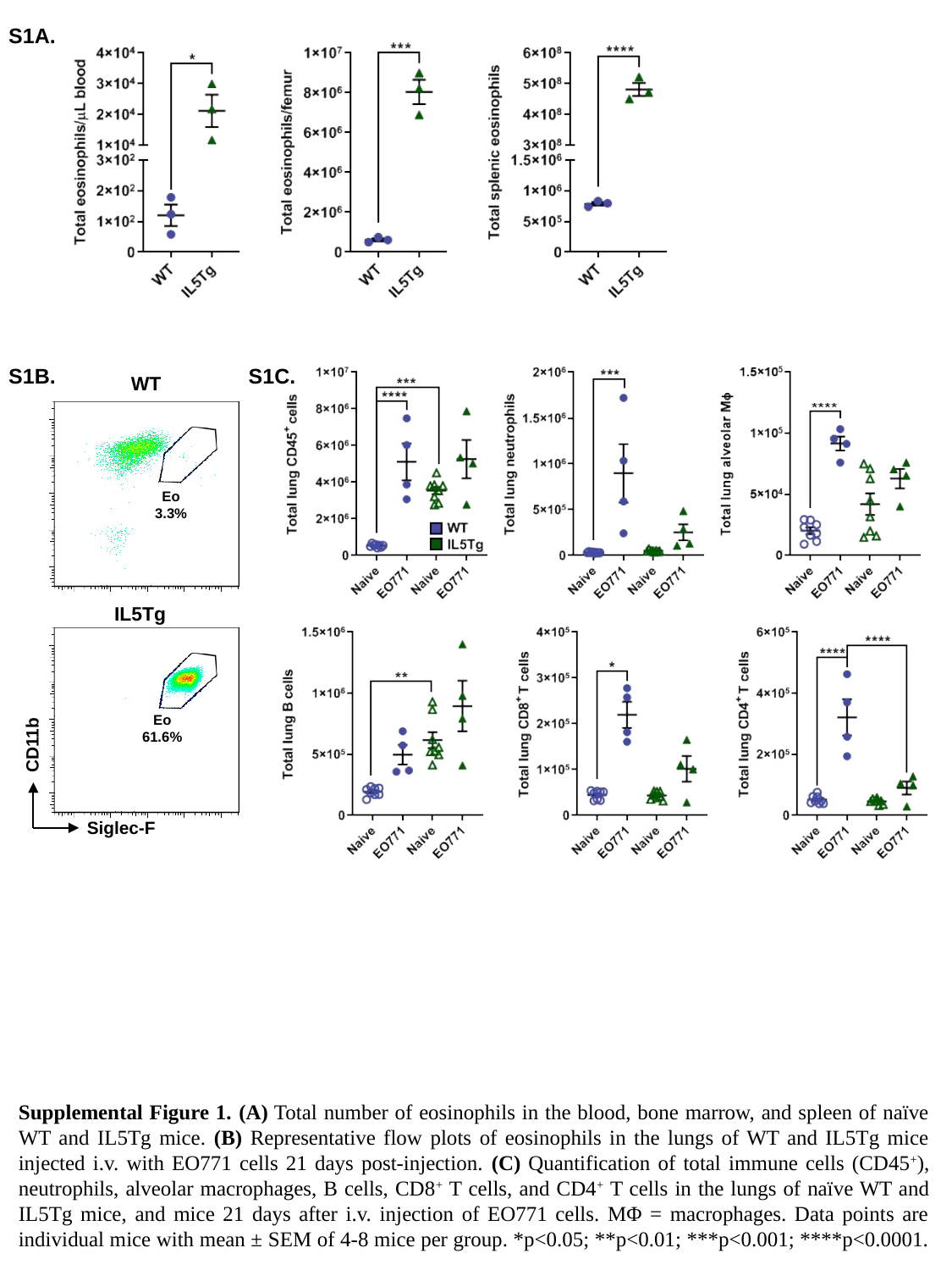

S1A.
S1B.
S1C.
WT
Eo
3.3%
IL5Tg
Eo
61.6%
CD11b
Siglec-F
Supplemental Figure 1. (A) Total number of eosinophils in the blood, bone marrow, and spleen of naïve WT and IL5Tg mice. (B) Representative flow plots of eosinophils in the lungs of WT and IL5Tg mice injected i.v. with EO771 cells 21 days post-injection. (C) Quantification of total immune cells (CD45+), neutrophils, alveolar macrophages, B cells, CD8+ T cells, and CD4+ T cells in the lungs of naïve WT and IL5Tg mice, and mice 21 days after i.v. injection of EO771 cells. MΦ = macrophages. Data points are individual mice with mean ± SEM of 4-8 mice per group. *p<0.05; **p<0.01; ***p<0.001; ****p<0.0001.

## Slide 2
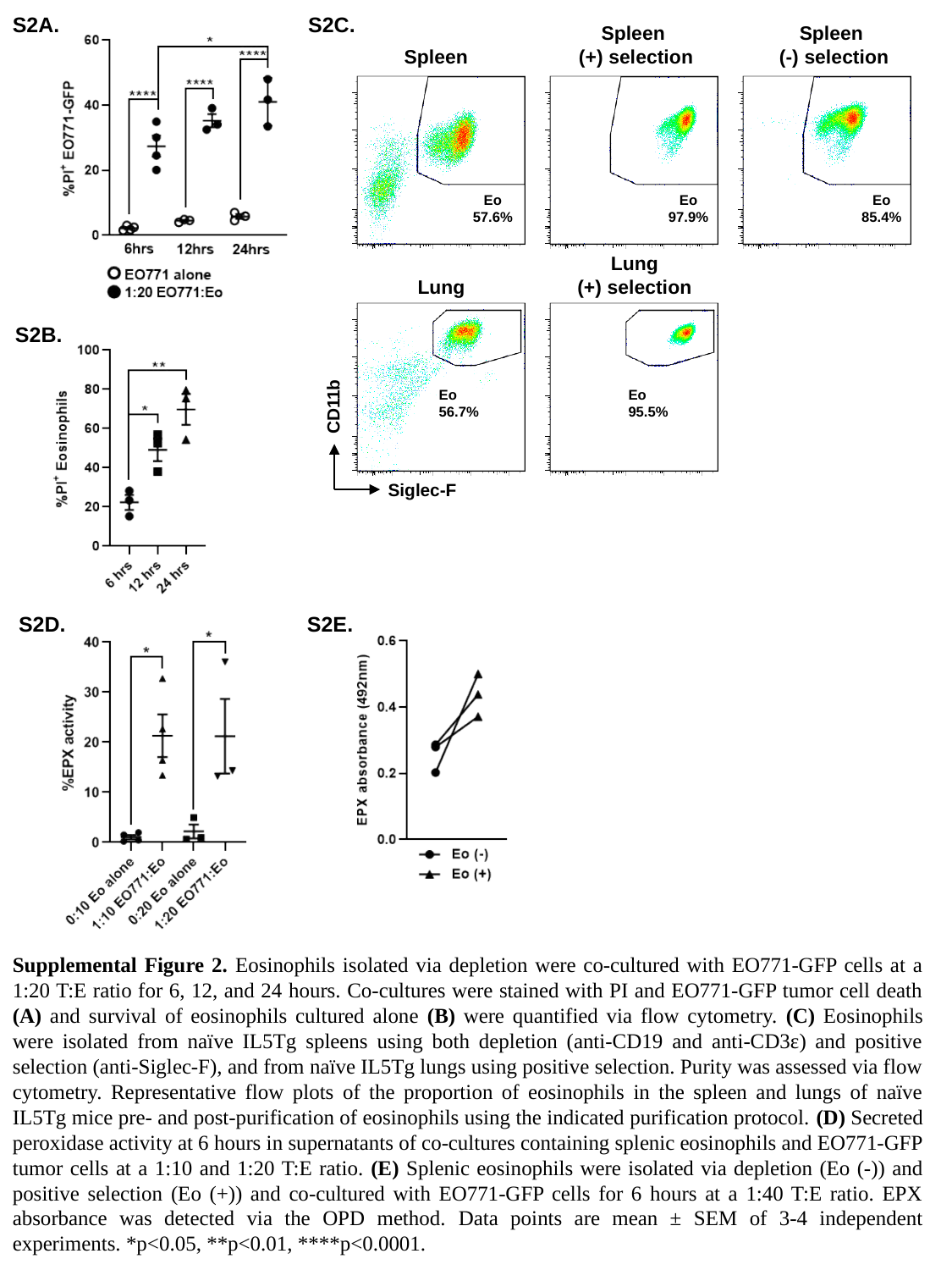

S2A.
S2C.
Spleen
(+) selection
Spleen
(-) selection
Spleen
Eo
85.4%
Eo
97.9%
Eo
57.6%
Lung
(+) selection
Lung
S2B.
Eo
95.5%
Eo
56.7%
CD11b
Siglec-F
S2D.
S2E.
Supplemental Figure 2. Eosinophils isolated via depletion were co-cultured with EO771-GFP cells at a 1:20 T:E ratio for 6, 12, and 24 hours. Co-cultures were stained with PI and EO771-GFP tumor cell death (A) and survival of eosinophils cultured alone (B) were quantified via flow cytometry. (C) Eosinophils were isolated from naïve IL5Tg spleens using both depletion (anti-CD19 and anti-CD3ε) and positive selection (anti-Siglec-F), and from naïve IL5Tg lungs using positive selection. Purity was assessed via flow cytometry. Representative flow plots of the proportion of eosinophils in the spleen and lungs of naïve IL5Tg mice pre- and post-purification of eosinophils using the indicated purification protocol. (D) Secreted peroxidase activity at 6 hours in supernatants of co-cultures containing splenic eosinophils and EO771-GFP tumor cells at a 1:10 and 1:20 T:E ratio. (E) Splenic eosinophils were isolated via depletion (Eo (-)) and positive selection (Eo (+)) and co-cultured with EO771-GFP cells for 6 hours at a 1:40 T:E ratio. EPX absorbance was detected via the OPD method. Data points are mean ± SEM of 3-4 independent experiments. *p<0.05, **p<0.01, ****p<0.0001.

## Slide 3
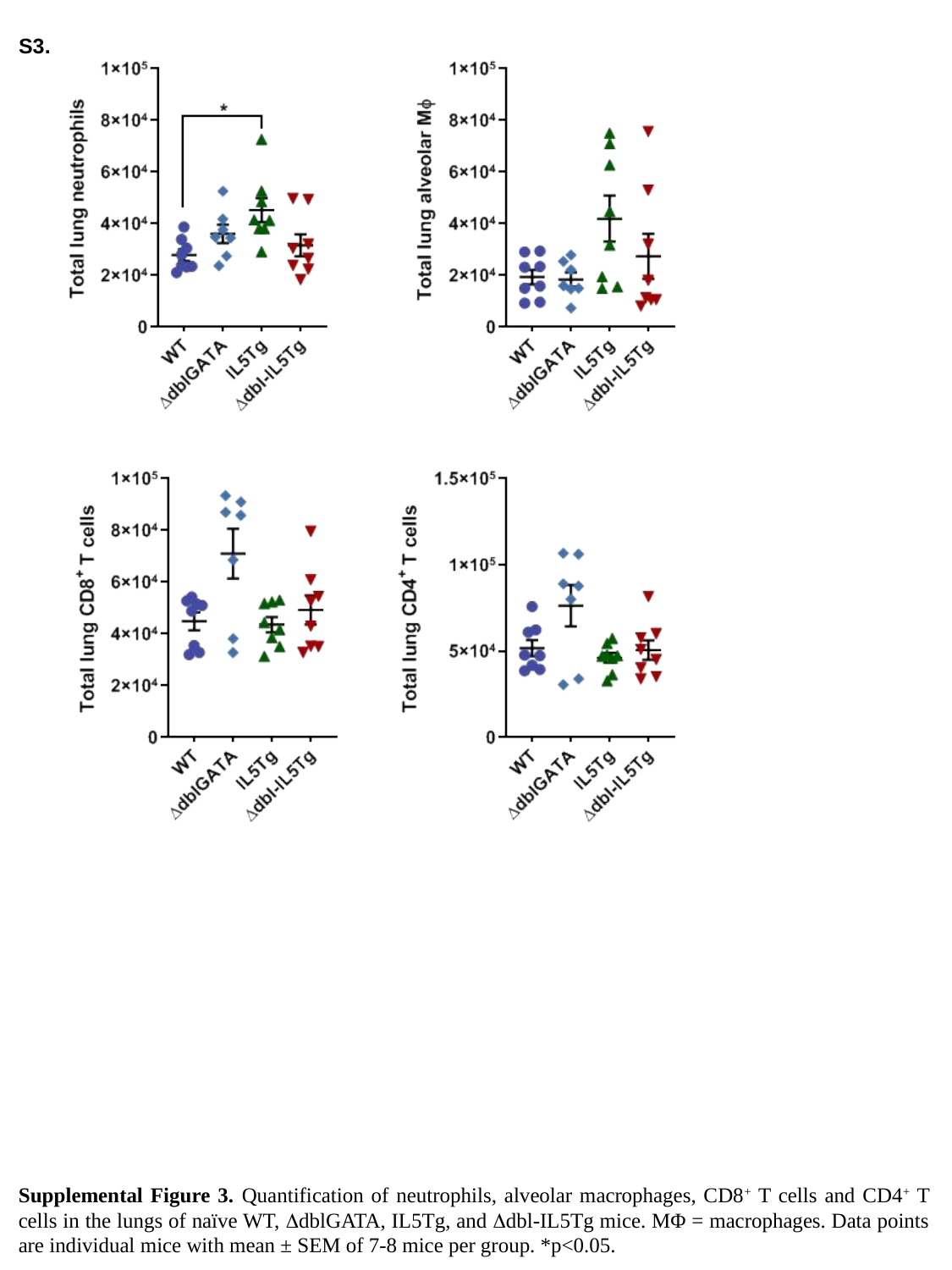

S3.
Supplemental Figure 3. Quantification of neutrophils, alveolar macrophages, CD8+ T cells and CD4+ T cells in the lungs of naïve WT, ΔdblGATA, IL5Tg, and Δdbl-IL5Tg mice. MΦ = macrophages. Data points are individual mice with mean ± SEM of 7-8 mice per group. *p<0.05.

## Slide 4
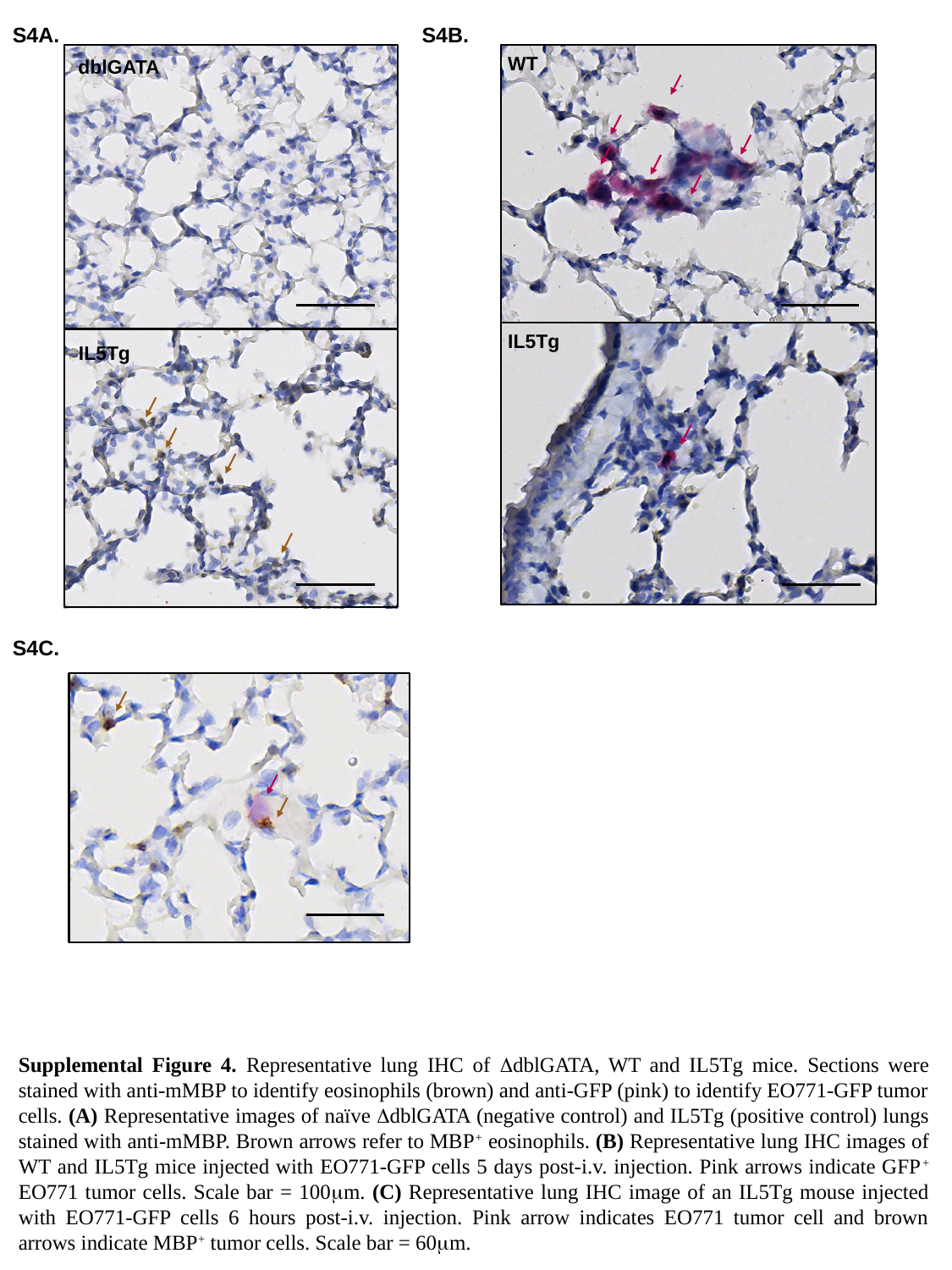

S4A.
S4B.
WT
IL5Tg
IL5Tg
S4C.
Supplemental Figure 4. Representative lung IHC of ΔdblGATA, WT and IL5Tg mice. Sections were stained with anti-mMBP to identify eosinophils (brown) and anti-GFP (pink) to identify EO771-GFP tumor cells. (A) Representative images of naïve ΔdblGATA (negative control) and IL5Tg (positive control) lungs stained with anti-mMBP. Brown arrows refer to MBP+ eosinophils. (B) Representative lung IHC images of WT and IL5Tg mice injected with EO771-GFP cells 5 days post-i.v. injection. Pink arrows indicate GFP+ EO771 tumor cells. Scale bar = 100μm. (C) Representative lung IHC image of an IL5Tg mouse injected with EO771-GFP cells 6 hours post-i.v. injection. Pink arrow indicates EO771 tumor cell and brown arrows indicate MBP+ tumor cells. Scale bar = 60μm.

## Slide 5
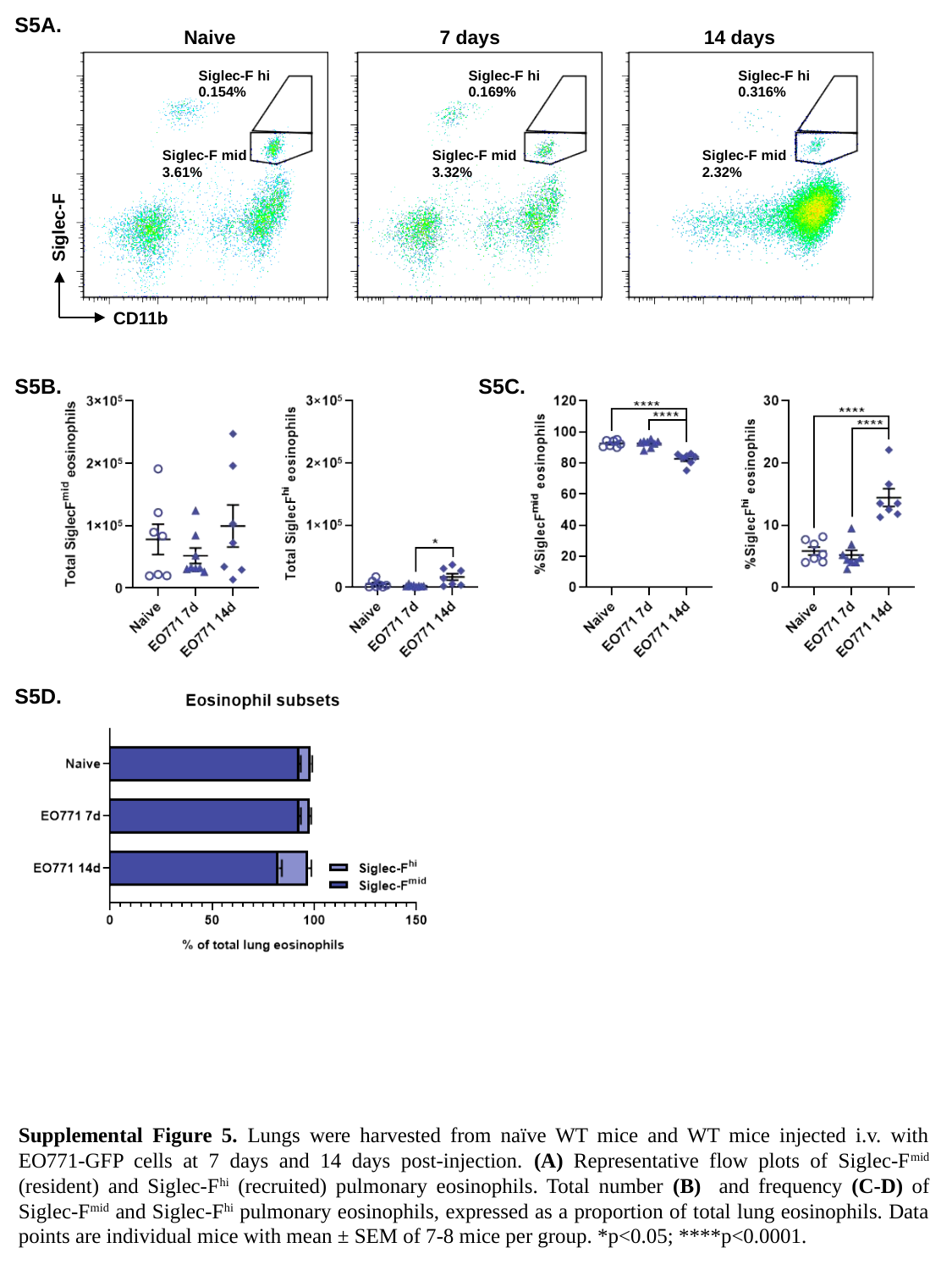

S5A.
Naive
7 days
14 days
Siglec-F hi
0.154%
Siglec-F hi
0.169%
Siglec-F hi
0.316%
Siglec-F mid
3.61%
Siglec-F mid
3.32%
Siglec-F mid
2.32%
Siglec-F
CD11b
S5B.
S5C.
S5D.
Supplemental Figure 5. Lungs were harvested from naïve WT mice and WT mice injected i.v. with EO771-GFP cells at 7 days and 14 days post-injection. (A) Representative flow plots of Siglec-Fmid (resident) and Siglec-Fhi (recruited) pulmonary eosinophils. Total number (B) and frequency (C-D) of Siglec-Fmid and Siglec-Fhi pulmonary eosinophils, expressed as a proportion of total lung eosinophils. Data points are individual mice with mean ± SEM of 7-8 mice per group. *p<0.05; ****p<0.0001.

## Slide 6
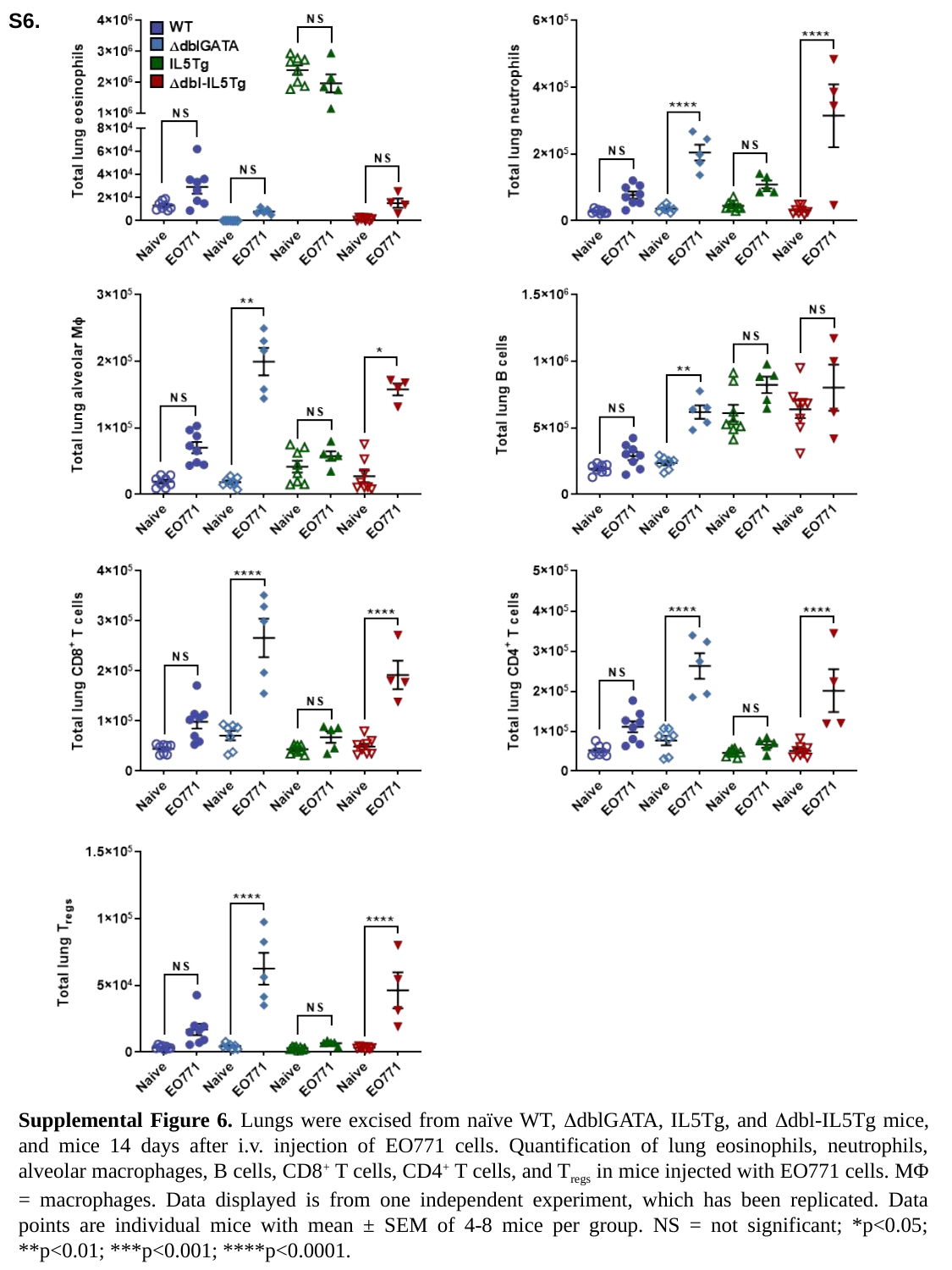

S6.
Supplemental Figure 6. Lungs were excised from naïve WT, ΔdblGATA, IL5Tg, and Δdbl-IL5Tg mice, and mice 14 days after i.v. injection of EO771 cells. Quantification of lung eosinophils, neutrophils, alveolar macrophages, B cells, CD8+ T cells, CD4+ T cells, and Tregs in mice injected with EO771 cells. MΦ = macrophages. Data displayed is from one independent experiment, which has been replicated. Data points are individual mice with mean ± SEM of 4-8 mice per group. NS = not significant; *p<0.05; **p<0.01; ***p<0.001; ****p<0.0001.

## Slide 7
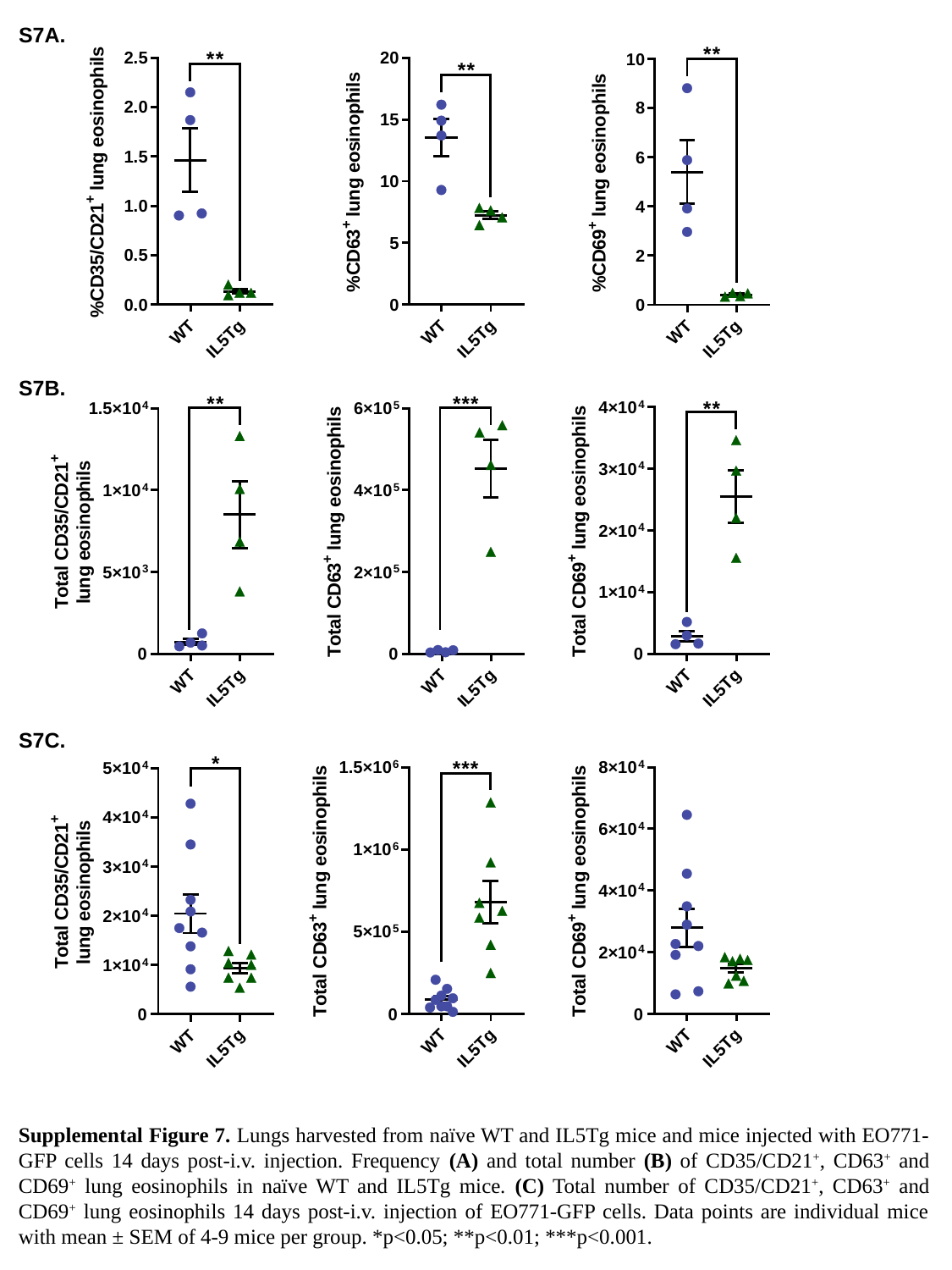

S7A.
S7B.
S7C.
Supplemental Figure 7. Lungs harvested from naïve WT and IL5Tg mice and mice injected with EO771-GFP cells 14 days post-i.v. injection. Frequency (A) and total number (B) of CD35/CD21+, CD63+ and CD69+ lung eosinophils in naïve WT and IL5Tg mice. (C) Total number of CD35/CD21+, CD63+ and CD69+ lung eosinophils 14 days post-i.v. injection of EO771-GFP cells. Data points are individual mice with mean ± SEM of 4-9 mice per group. *p<0.05; **p<0.01; ***p<0.001.

## Slide 8
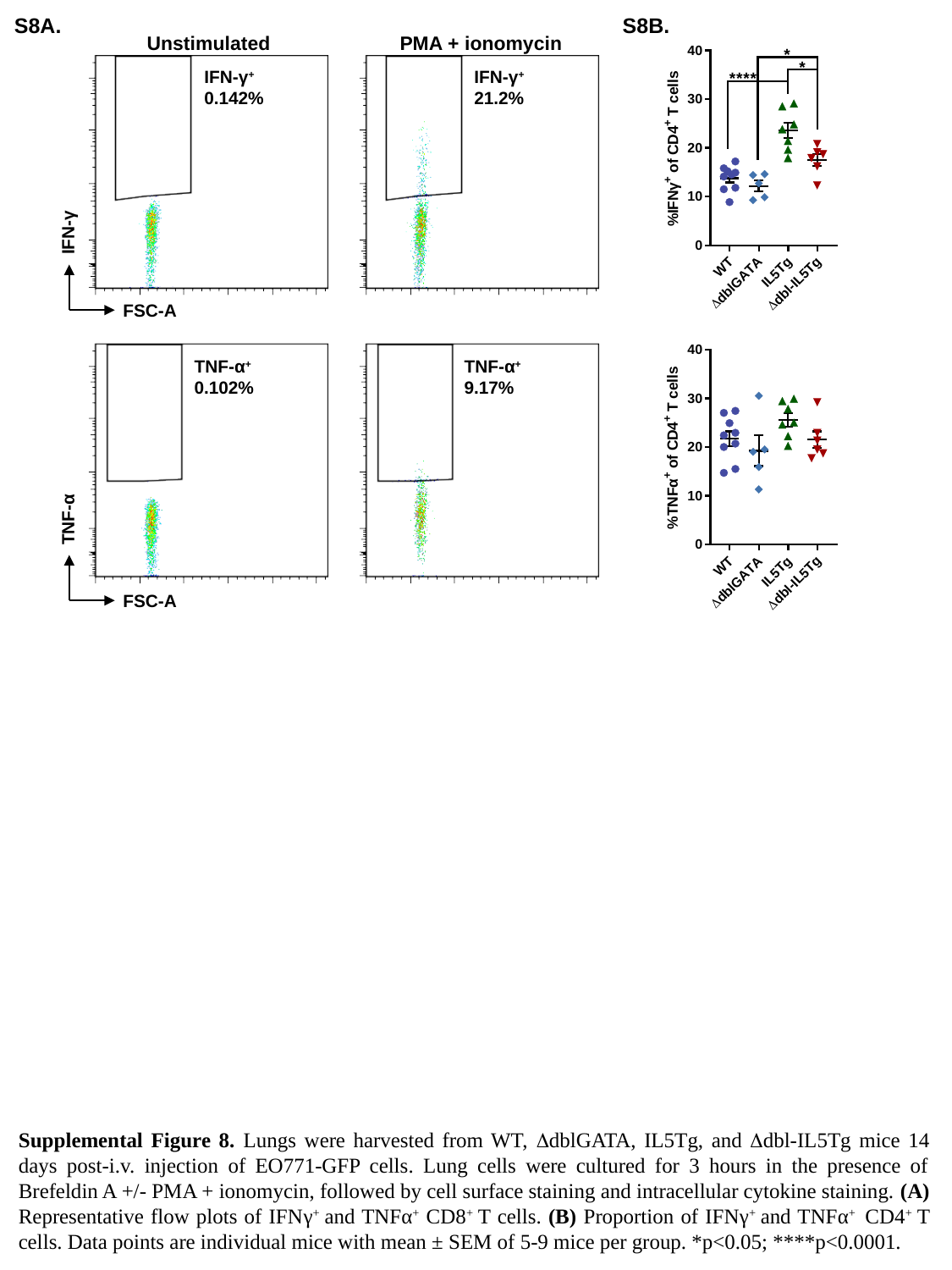

S8A.
S8B.
Unstimulated
PMA + ionomycin
IFN-γ+
0.142%
IFN-γ+
21.2%
IFN-γ
FSC-A
TNF-α+
0.102%
TNF-α+
9.17%
TNF-α
FSC-A
Supplemental Figure 8. Lungs were harvested from WT, ΔdblGATA, IL5Tg, and Δdbl-IL5Tg mice 14 days post-i.v. injection of EO771-GFP cells. Lung cells were cultured for 3 hours in the presence of Brefeldin A +/- PMA + ionomycin, followed by cell surface staining and intracellular cytokine staining. (A) Representative flow plots of IFNγ+ and TNFα+ CD8+ T cells. (B) Proportion of IFNγ+ and TNFα+ CD4+ T cells. Data points are individual mice with mean ± SEM of 5-9 mice per group. *p<0.05; ****p<0.0001.

## Slide 9
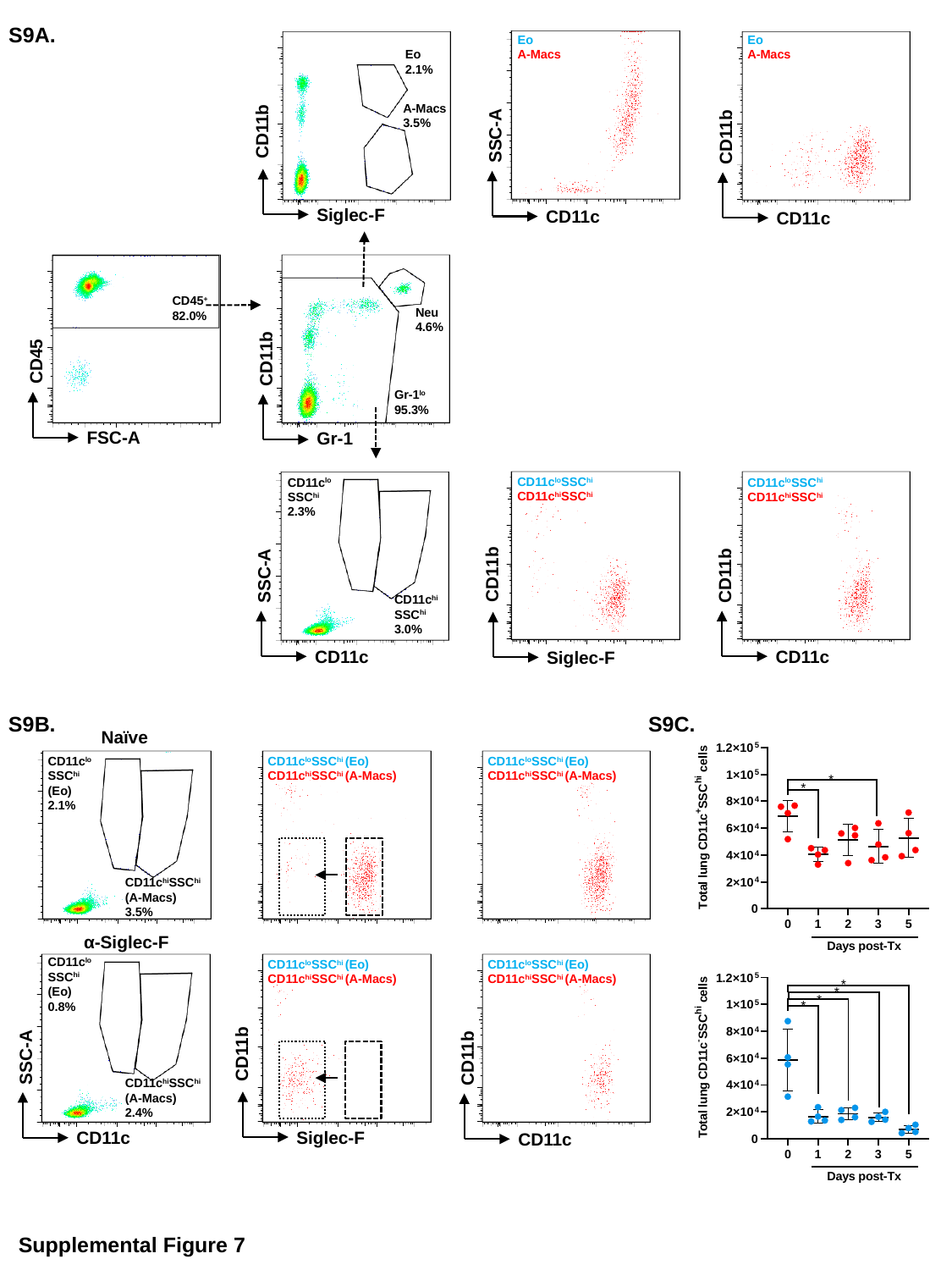

S9A.
Eo
A-Macs
Eo
A-Macs
Eo
2.1%
A-Macs
3.5%
CD11b
SSC-A
CD11b
Siglec-F
CD11c
CD11c
CD45+
82.0%
Neu
4.6%
CD45
CD11b
Gr-1lo
95.3%
FSC-A
Gr-1
CD11cloSSChi
CD11chiSSChi
CD11clo
SSChi
2.3%
CD11cloSSChi
CD11chiSSChi
CD11b
CD11b
SSC-A
CD11chi
SSChi
3.0%
CD11c
CD11c
Siglec-F
S9B.
Naïve
CD11clo
SSChi
(Eo)
2.1%
CD11cloSSChi (Eo)
CD11chiSSChi (A-Macs)
CD11cloSSChi (Eo)
CD11chiSSChi (A-Macs)
CD11chiSSChi
(A-Macs)
3.5%
α-Siglec-F
CD11clo
SSChi
(Eo)
0.8%
CD11cloSSChi (Eo)
CD11chiSSChi (A-Macs)
CD11cloSSChi (Eo)
CD11chiSSChi (A-Macs)
CD11b
SSC-A
CD11b
CD11chiSSChi
(A-Macs)
2.4%
Siglec-F
CD11c
CD11c
S9C.
Supplemental Figure 7

## Slide 10
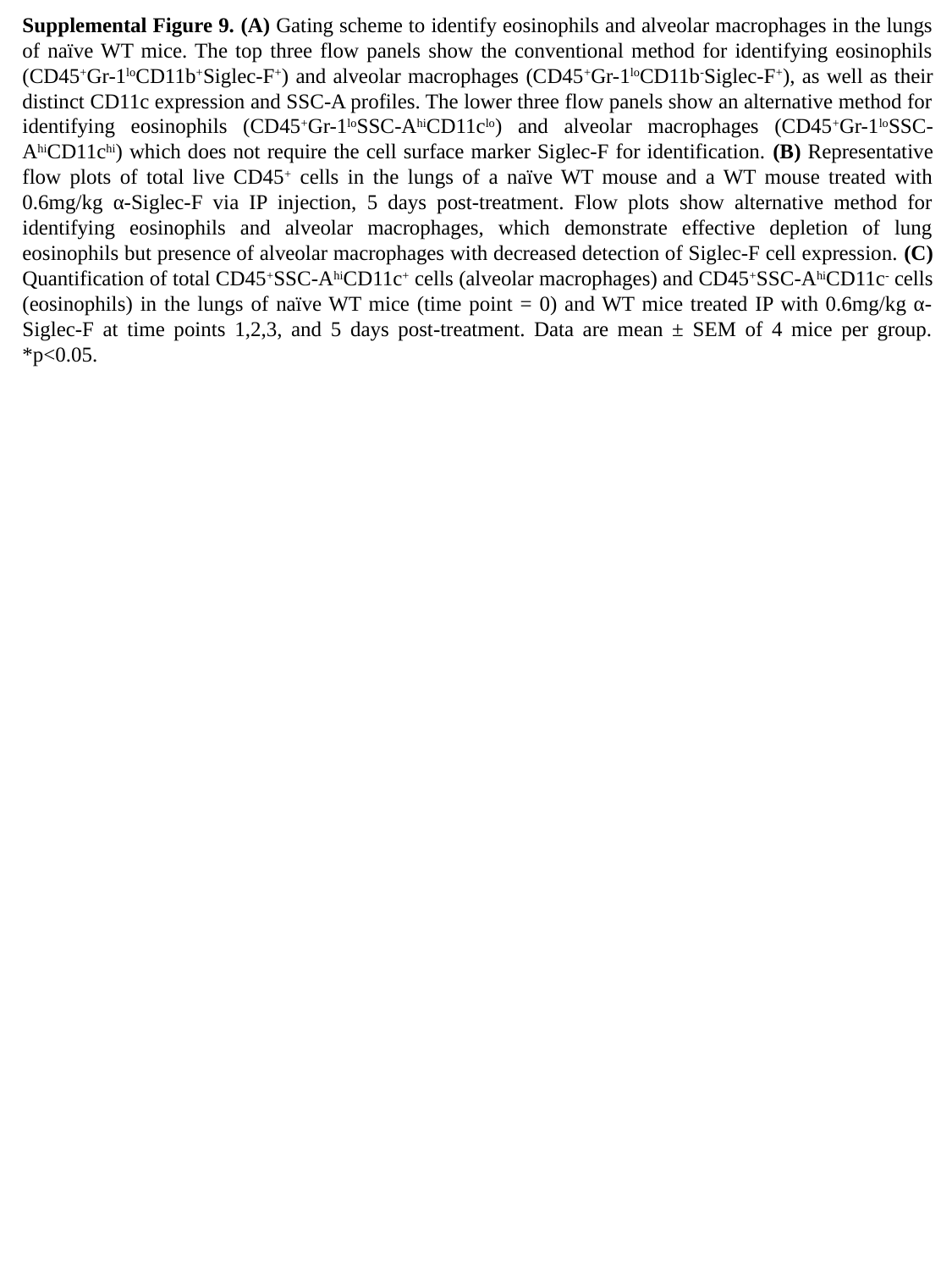

Supplemental Figure 9. (A) Gating scheme to identify eosinophils and alveolar macrophages in the lungs of naïve WT mice. The top three flow panels show the conventional method for identifying eosinophils (CD45+Gr-1loCD11b+Siglec-F+) and alveolar macrophages (CD45+Gr-1loCD11b-Siglec-F+), as well as their distinct CD11c expression and SSC-A profiles. The lower three flow panels show an alternative method for identifying eosinophils (CD45+Gr-1loSSC-AhiCD11clo) and alveolar macrophages (CD45+Gr-1loSSC-AhiCD11chi) which does not require the cell surface marker Siglec-F for identification. (B) Representative flow plots of total live CD45+ cells in the lungs of a naïve WT mouse and a WT mouse treated with 0.6mg/kg α-Siglec-F via IP injection, 5 days post-treatment. Flow plots show alternative method for identifying eosinophils and alveolar macrophages, which demonstrate effective depletion of lung eosinophils but presence of alveolar macrophages with decreased detection of Siglec-F cell expression. (C) Quantification of total CD45+SSC-AhiCD11c+ cells (alveolar macrophages) and CD45+SSC-AhiCD11c- cells (eosinophils) in the lungs of naïve WT mice (time point = 0) and WT mice treated IP with 0.6mg/kg α-Siglec-F at time points 1,2,3, and 5 days post-treatment. Data are mean ± SEM of 4 mice per group. *p<0.05.

## Slide 11
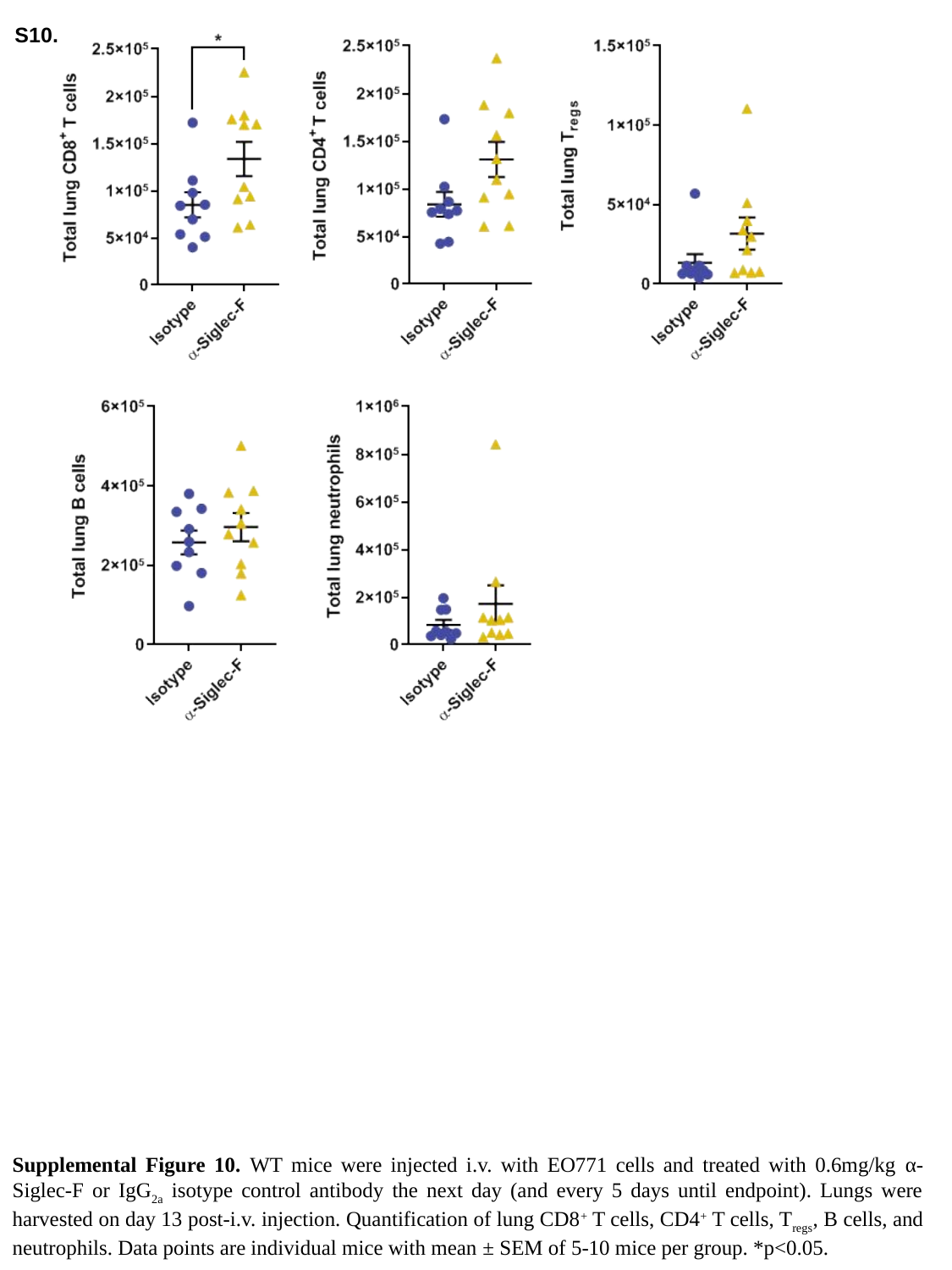

S10.
Supplemental Figure 10. WT mice were injected i.v. with EO771 cells and treated with 0.6mg/kg α-Siglec-F or IgG2a isotype control antibody the next day (and every 5 days until endpoint). Lungs were harvested on day 13 post-i.v. injection. Quantification of lung CD8+ T cells, CD4+ T cells, Tregs, B cells, and neutrophils. Data points are individual mice with mean ± SEM of 5-10 mice per group. *p<0.05.
